# Review on the Synthesis and Therapeutic Potential of Pyrido[2,3-*d*], [3,2-*d*], [3,4-*d*] and [4,3-*d*]pyrimidine Derivatives

**DOI:** 10.3390/ph15030352

**Published:** 2022-03-14

**Authors:** Joana F. Campos, Thierry Besson, Sabine Berteina-Raboin

**Affiliations:** 1 Institut de Chimie Organique et Analytique (ICOA), Université d’Orléans CNRS, ICOA UMR 7311, BP 6759, Rue de Chartres, CEDEX 2, 45067 Orléans, France; joanafilomenamsc@gmail.com; 2 Université de Rouen-Normandie (UNIROUEN), INSA Rouen, CNRS, COBRA UMR 6014, 76000 Rouen, France; thierry.besson@univ-rouen.fr

**Keywords:** pyridopyrimidines, synthesis, biological activity, *N*-heterocycles

## Abstract

The objective of this review is to list the structures composed of a pyridopyrimidine moiety which have shown a therapeutic interest or have already been approved for use as therapeutics. We consider all the synthetic protocols to prepare these pyridopyrimidine derivatives. The review is organized into four sections, successively pyrido[2,3-*d*]pyrimidines, pyrido[3,4-*d*]pyrimidines, pyrido[4,3-*d*]pyrimidines and pyrido[3,2-*d*]pyrimidines. For each compound we present the biological activity and the synthetic route reported. To produce this manuscript, the bibliographic research was done using Reaxys and Scifinder for each kind of pyridopyrimidine.

## 1. Introduction: Pyridopyrimidines and Their Scaffold

**Figure 1:**
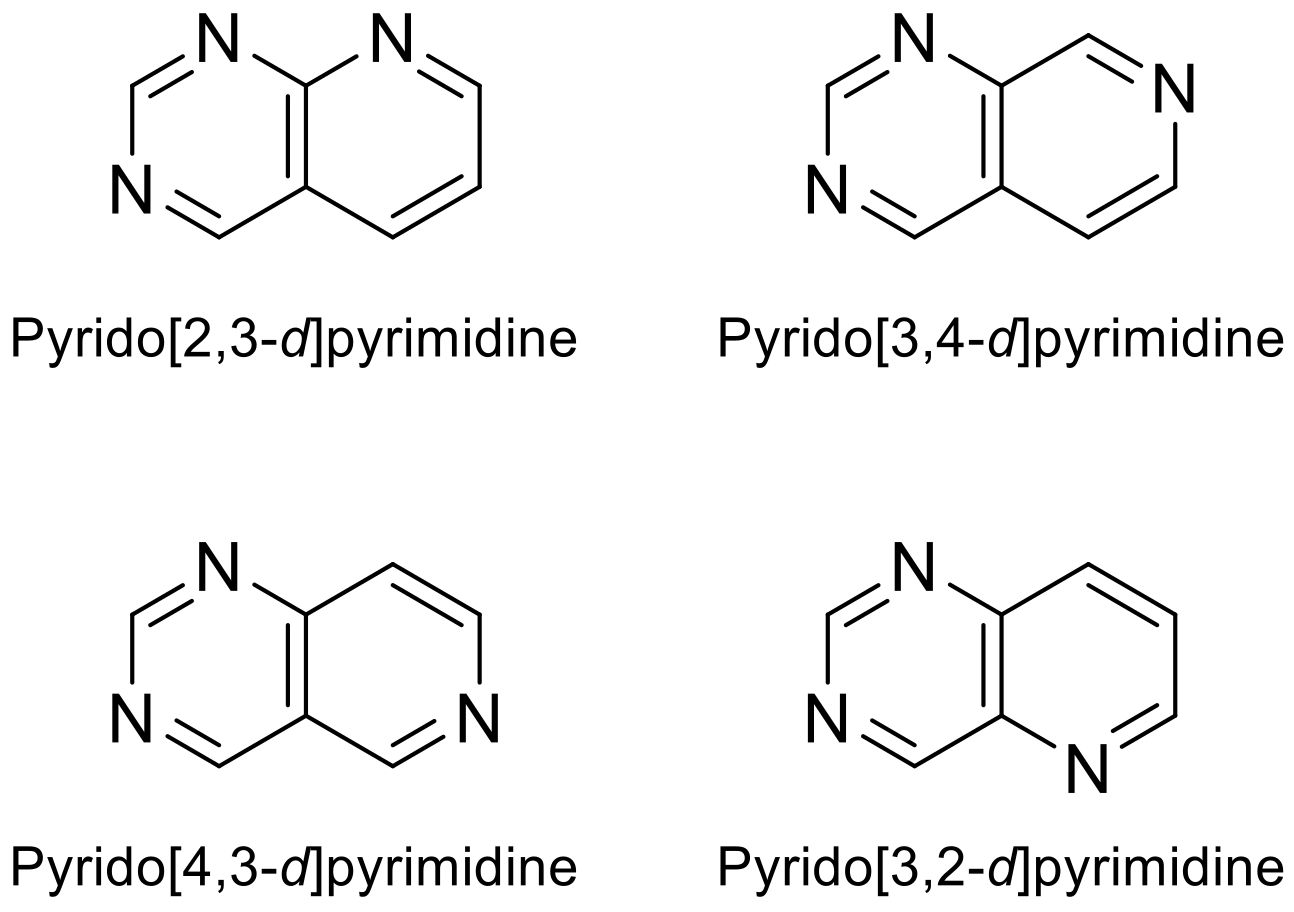
Various pyridopyrimidine structures types.

Depending on where the nitrogen atom is located in pyridine, we can find four possible skeletons for the heterocyclic combination of pyrimidine and pyridine rings (Figure 1). Pyridopyrimidines and other *N*-heterocycles are of great interest due to their biological potential. The pyridopyrimidine moiety is present in relevant drugs and, in recent years, it has been studied in the development of new therapies, as evidenced by numerous publications, studies and clinical trials [1,2,3].

**Figure 2:**
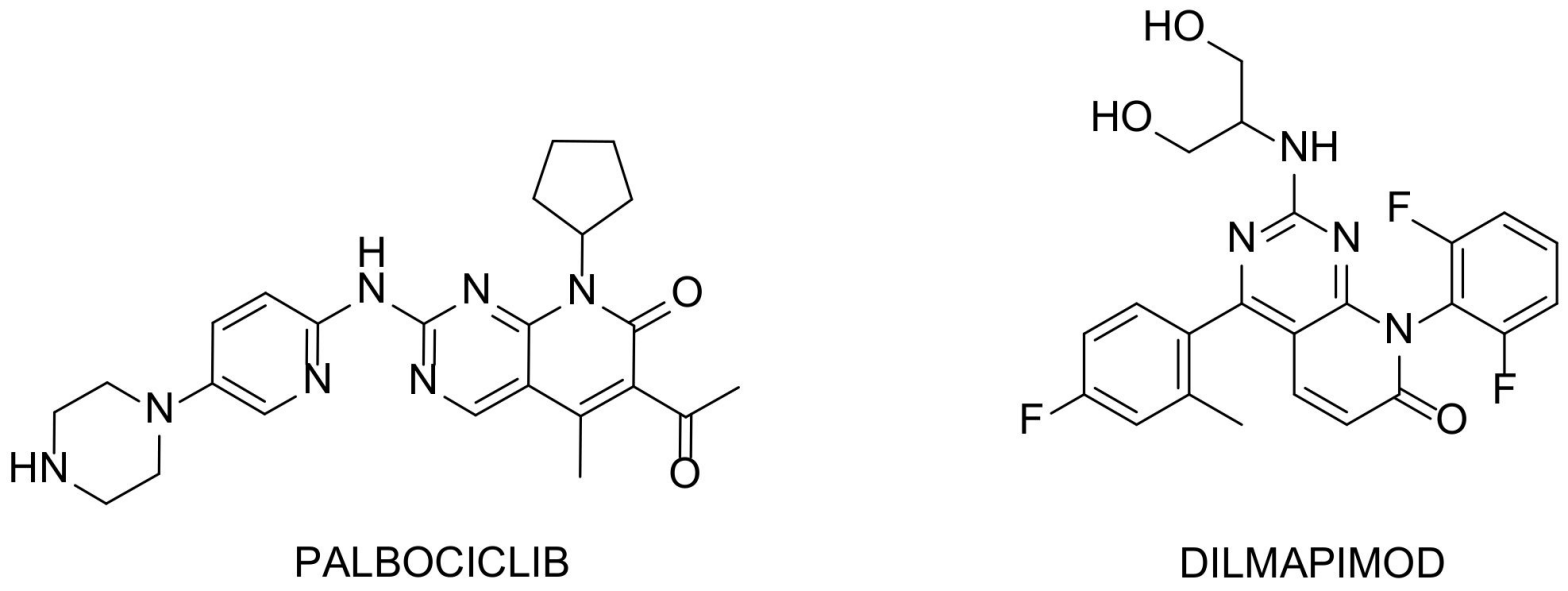
Examples of interesting molecules. Palbociclib: breast cancer drug developed by Pfizer and Dilmapimod: potential activity against rheumatoid arthritis.

The various pyridopyrimidines are used on several therapeutic targets. We consider all the synthetic protocols to prepare these pyridopyrimidine derivatives which have shown a therapeutic interest or have been approved for use as therapeutics according to bibliographic research conducted on Reaxys and Scifinder. Among them, we can mention in Figure 2 palbociclib and dilmapimod.

Those most frequently mentioned biological targets of pyrido[2,3-d]pyrimidine derivatives are dihydrofolate reductase (DHFR), some kinases, such as the tyrosine-protein kinase transforming protein Abl or MAP kinases, and the biotin carboxylase.

Dihydrofolate reductase (DHFR) catalyzes the reduction of dihydrofolate to tetrahydrofolate which is essential for the action of folate-dependent enzymes and then for DNA synthesis and methylation reactions. This enzyme is very important for converting the inactive form of folic acid into an active form, which is crucial to make some building blocks required for DNA production. By inhibiting this enzyme, the drug affects the capacity of cells to repair and replicate. The pyridopyrimidine drug inhibits DHFR with high affinity, thereby reducing the quantity of tetrahydrofolate necessary for the synthesis of pyrimidine and purine. Therefore, the synthesis of RNA and DNA is stopped, and the cancer cells die [4,5]. Inhibitors of this enzyme are studied and used in the treatment of several diseases such as psoriasis and autoimmune rhumatoid arthritis, to name but two. A fuller list can be found in [6,7].

Kinases or protein kinases are the generic names of enzymes involved in the signaling pathways that preside over a large number of cellular functions and are involved in various pathologies, including cancerous pathologies [8,9,10,11]. For example, tyrosine protein kinase plays a role in many key processes linked to cell growth and survival.

Pyridopyrimidines are kinase inhibitors and act by competition on the active site or at an allosteric site. Various tyrosine kinase inhibitors, called tyrphostines (e.g., imatinib, gefitinib, sunitinib), which act selectively on one or more receptors with tyrosine kinase activity, are used to treat some specific forms of cancer.

While many inhibitors have already showed great therapeutic potential, intensive research effort is currently underway to discover new molecules able to interact with protein kinases for use in therapy.

Biotin dependent carboxylases can be found in numerous species of fungi, bacteria, plants and, of course, animals and humans. They play an important role in various metabolisms such as fatty acids [12], carbohydrates and amino acids, but also assimilation [13,14,15,16,17,18,19] and fixation [20]. Biotin dependent carboxylases contain acetyl-CoA carboxylase (ACC), propionyl-CoA carboxylase (PCC), 3-methylcrotonyl-CoA carboxylase (MCC), geranyl-CoA carboxylase (GCC), pyruvate carboxylase (PC), and urea carboxylase (UC). Due to their activity, they are mainly involved in diseases such as type 2 diabetes, obesity and microbial infection [21]. ACC catalyzes the carboxylation of acetyl-CoA to form malonyl-CoA, which is an intermediate substrate. Over the years, ACC inhibitors have attracted great attention in the development of treatments for various human diseases, including microbial infections, metabolic syndrome, obesity, diabetes and cancer [22,23].

## 2. Pyridopyrimidines: Therapeutic Potential and Synthesis

In this section, we describe, for each compound mentioned, the biological activity and the synthetic route reported. The 24 compounds described herein are presented according to the type of pyridopyrimidines (pyrido[2,3-*d*]pyrimidine, pyrido[3,4-*d*]pyrimidine, pyrido[4,3-*d*]pyrimidine and pyrido[3,2-*d*]pyrimidine). For each compound described, the target is indicated and some additional information has been added if different from that mentioned in the introduction.

### 2.1. Pyrido[2,3-d]pyrimidine

The study starts with some interesting pyrido[2,3-*d*]pyrimidines. The first one is 5-methyl-6-([methyl(3,4,5-trimethoxyphenyl)amino]methyl)pyrido[2,3-*d*]pyrimidine-2,4-diamine (Table 1, entry 1) which has been described to have DHFR dihydrofolate as the target [6].

**Scheme 1:**
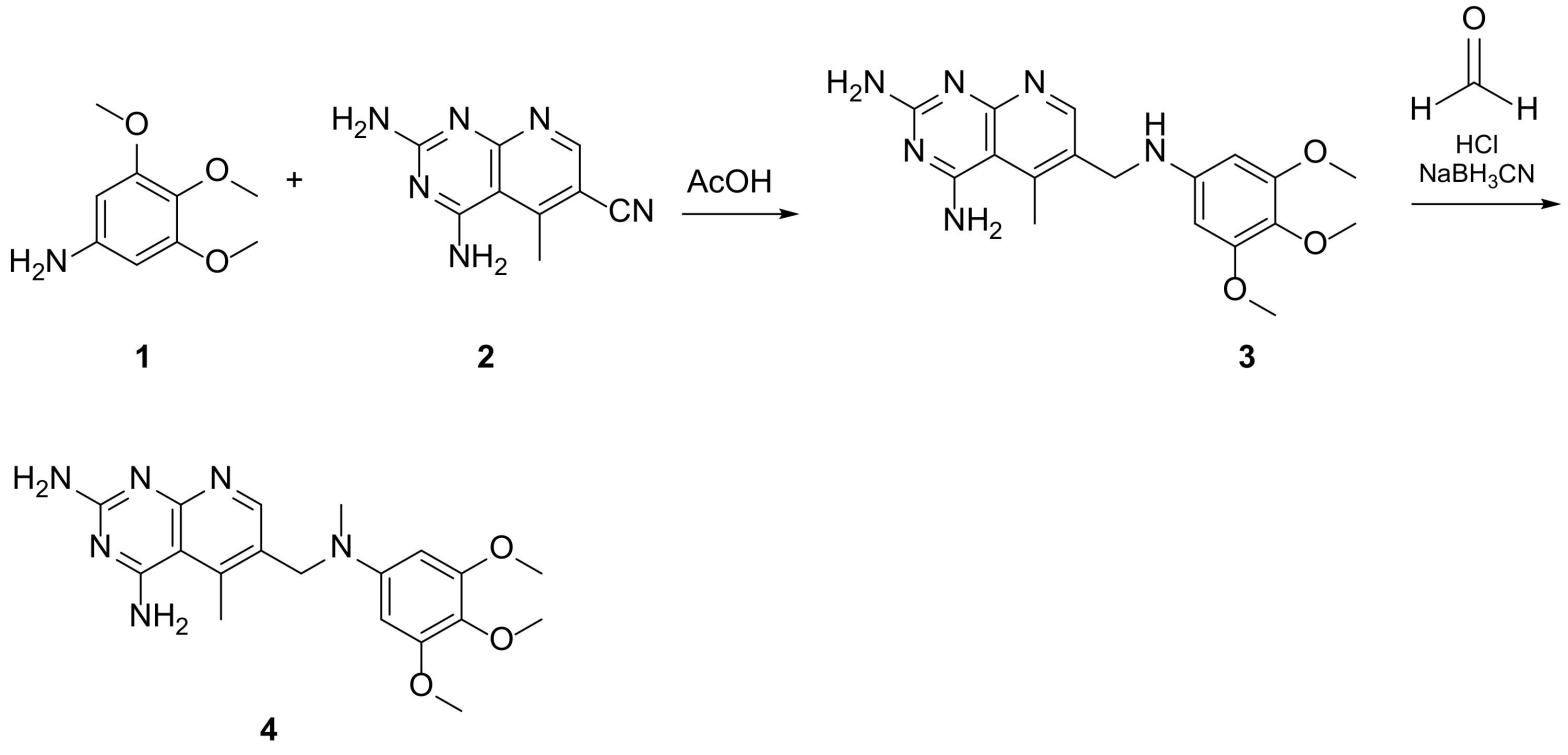
Synthesis of pyrido[2,3-*d*]pyrimidine-2,4-diamine (**4**) by Kisliuk et al. [6].

Kisliuk et al. described, in 1993, the synthesis of pyrido[2,3-*d*]pyrimidine-2,4-diamine (**4**). The reductive condensation of 6-cyano-5-methyl-pyrido[2,3-*d*]pyrimidine-2,4-diamine (**2**) with 3,4,5-trimethoxyaniline (**1**) in the presence of Raney Ni 70% in acetic acid gave the precursor **3** which underwent methylation at the N10 position by reductive alkylation with formaldehyde and sodium cyanoborohydride (Scheme 1) [6].

**Scheme 2:**
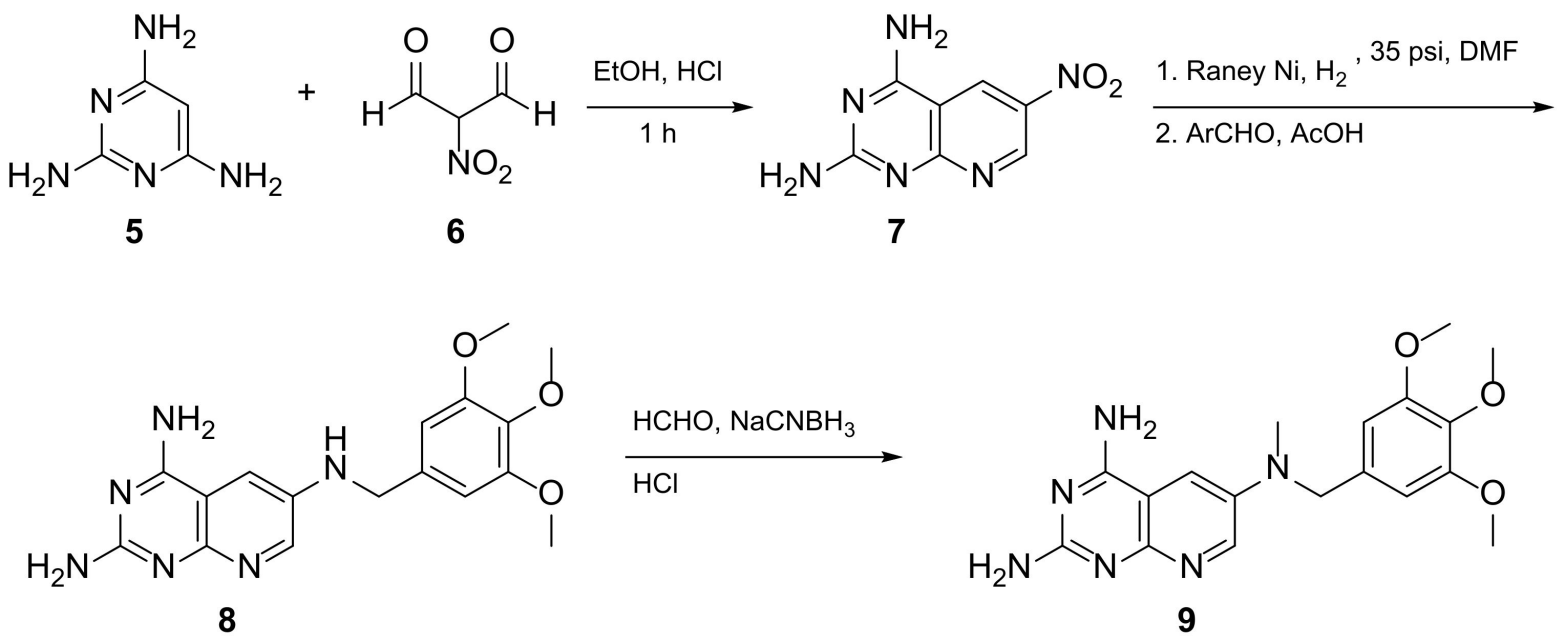
Synthesis of pyrido[2,3-*d*]pyrimidine-2,4-diamine (**9**) by Kisliuk et al. [19].

Kisliuk et al. also developed another strategy to synthesize pyrido[2,3-*d*] pyrimidine-2,4-diamines as compound **9** (Scheme 2, Table 1, entry 2). Starting from 2,4,6-triaminopyrimidine (**5**) with the sodium salt of nitromalonaldehyde, they obtained in a single step the 2,4-diamino-6-nitropyrido [2,3-*d*]pyrimidine (**7**) which was then reduced to its corresponding 6-amino analogue using Raney Ni in DMF. The reductive amination with various aldehydes (ArCHO, in this case 3,4,5-trimethoxybenzaldehyde) provided the desired product **8**. In the last step, **8** was *N*-methylated by treatment with formaldehyde in the presence of sodium cyanoborohydride [19] (Scheme 2). An analog compound (Table 1, entry 3) was obtained following the same synthetic pathway (Scheme 2) using 3,5-dimethoxybenzaldehyde.

**Scheme 3:**
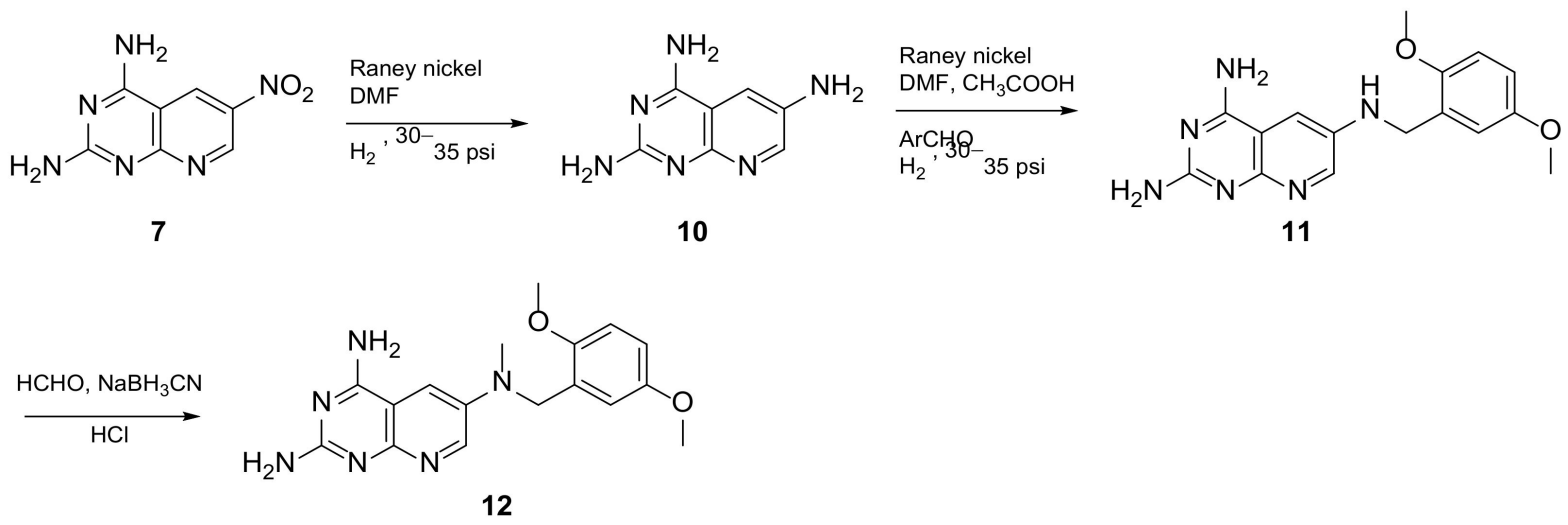
Synthesis of *N*6-[(2,5-dimethoxyphenyl)methyl]-*N*6-methylpyrido[2,3-*d*]pyrimidine-2,4,6-triamine (**12**) by Queener et al. [18].

In 2008, Queener et al. synthesized **12** starting from 2,4-diamino-6-nitroquinazoline **7** which underwent reduction with hydrogen and Raney nickel at 30-35 psi, providing the desired 2,4,6-triaminoquinazoline (**10**) (Scheme 3). Then, as described above, the 2,5-dimethoxybenzaldehyde ArCHO was added to generate the N9-H precursor **11**. The following step was a reductive N9-alkylation using sodium cyanoborohydride which afforded the final compound [18]. In this study, Queener et al. conducted a biological evaluation of this compound **12** (Scheme 3, Table 1, entry 4) as a lipophilic inhibitor of dihydrofolate reductase.

**Scheme 4:**
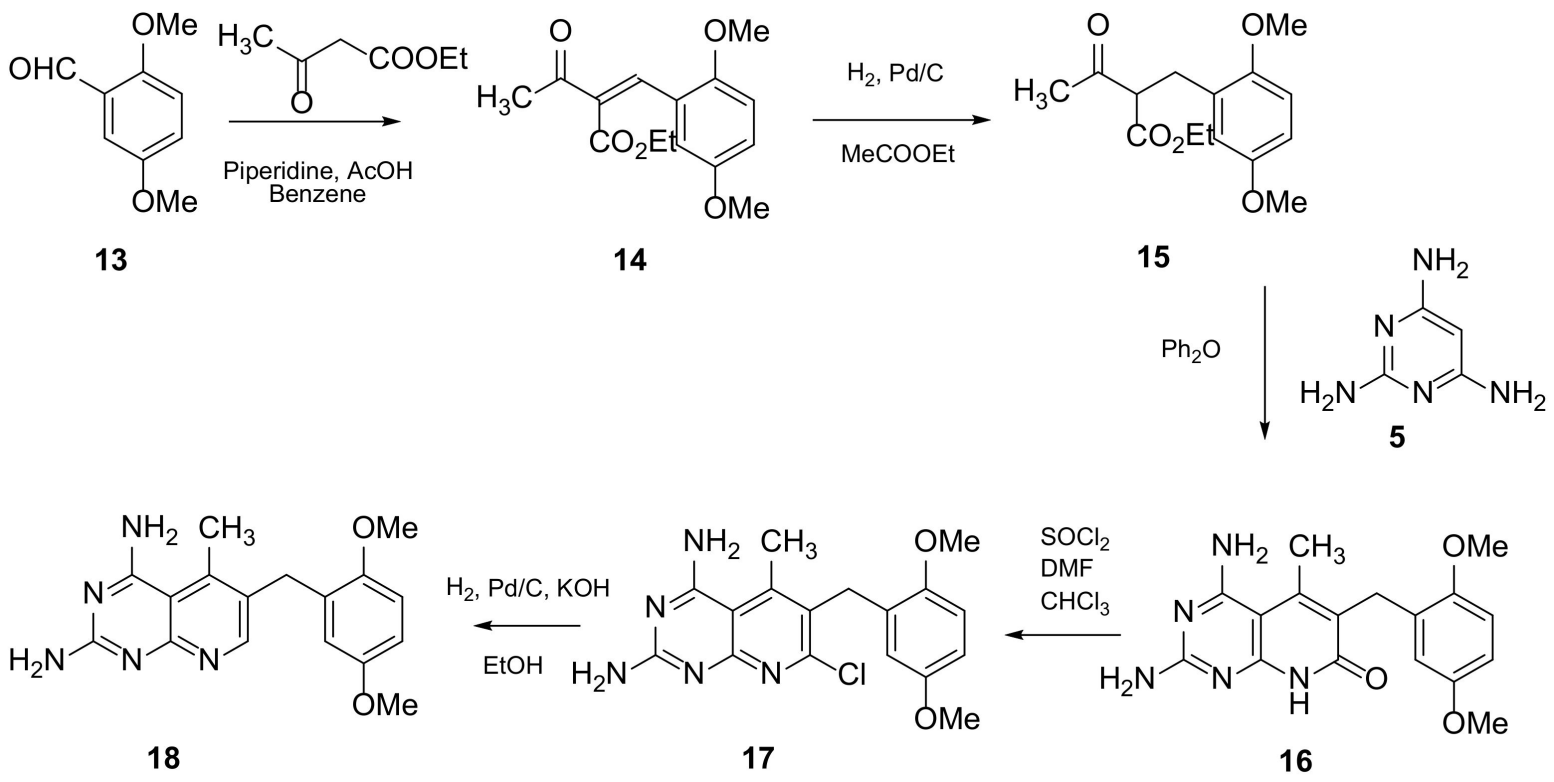
Synthesis of 6-[(2,5-dimethoxyphenyl)methyl]-5-methylpyrido[2,3-*d*]pyrimidine-2,4-diamine (**18**) by Grivsky, Sigel et al. [24].

**Scheme 5:**
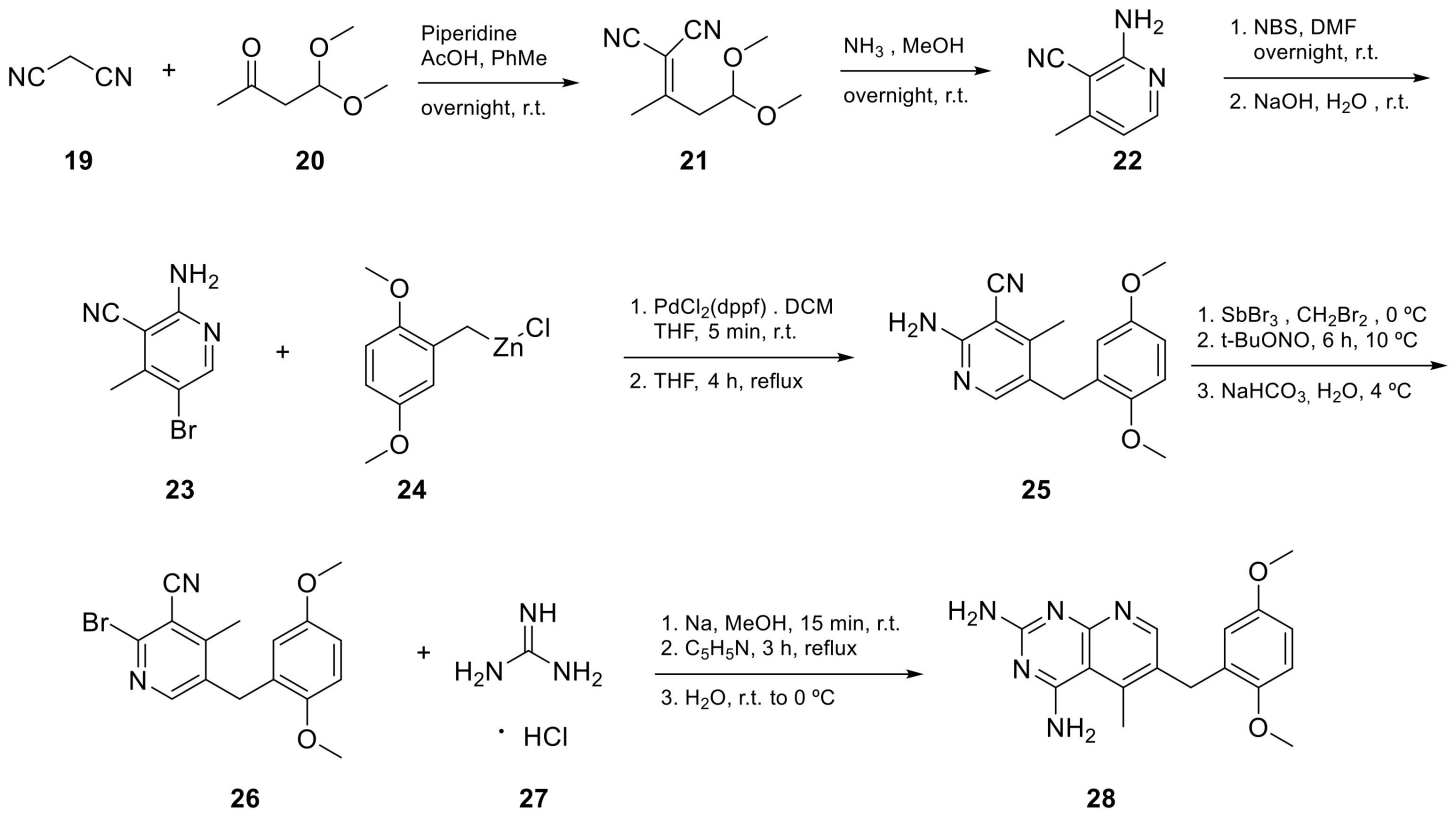
Synthesis of 6-[(2,5-dimethoxyphenyl)methyl]-5-methylpyrido[2,3-*d*]pyrimidine-2-amine (**28**) by Chan and Rosowsky [21].

Piritrexim (PTX) (Scheme 4 and Scheme 5, Table 1, entry 5) is a synthetic antifolate first synthesized by Grivsky, Sigel et al. [24] with anti-parasitic, anti-psoriatic and anti-tumor properties. Piritrexim inhibited dihydrofolate reductase (DHFR) and also showed good antitumor effects on the carcinosarcoma in rats. An advantage of this compound compared to some analogues is that it does not have effects as an inhibitor of histamine metabolism, reducing the potential risk of side reactions on metabolism. Its degree of lipophilicity, i.e., the affinity of this drug for a lipid environment, allows it to diffuse easily into the cells. The various therapeutical activities listed for piritrexim are on melanoma and urothelial cancer, and promising results in head and neck cancer were already obtained in combination with other molecules [20].

The reaction of 2,5-dimethoxybenzaldehyde (**13**) with ethyl acetoacetate in refluxing benzene in the presence of a mixture of piperidine and glacial acetic acid led to ethyl α-acetyl-β-(2,5-dimethoxyphenyl)acrylate (**14**). The latter is then hydrogenated using 5% Pd/C as a catalyst to yield the desired ethyl α-acetyl-(2,5-dimethoxyphenyl)propionate (**15**). Then, **15** was condensed with 2,4.6-triaminopyrimidine in a diphenyl ether at 195–230 °C. The 2,4-diamino-7,8-dihydro-6-(2,5-dimethoxybenzyl)-5-methyl-7-oxopyrido[2,3-*d*]pyrimidine (**16**) obtained was treated with a 1:1 complex of *N*,*N*-dimethylformamide thionyl chloride to give the expected 7-chloro-6-[(2,5-dimethoxyphenyl)methyl]-5-methylpyrido[2,3-*d*]pyrimidine-2,4-diamine (**17**). The desired PTX **18** was obtained by the hydrogenolyzation of **17** using Pd/C in the presence of potassium hydroxide (Scheme 4) [24].

Chan and Rosowsky reported the synthesis of PTX using the condensation of 4,4-dimethoxy-2-butanone with malononitrile to obtain the ylidenemalononitrile **21** which was cyclized into 2-amino-4-methyl-3-carbonitrilepyridine (**22**) using ammonia in methanol. The halogenation of **22** with *N*-bromosuccinimide in DMF gave the 5-bromo derivative **23**. The reaction of **23** in dry THF with 2,5-dimethoxybenzyl- zinc chloride and CH_2_Cl_2_ [PdCl_2_(dppf).CH_2_Cl_2_] provided 2-amino-5-(2,5-dimethoxybenzyl)-4-methyl-3-carbonitrilepyridine (**25**). Subsequently, the nonaqueous diazotization of **25** with t-BuONO and SbBr_3_ in CH_2_Br_2_ gave the bromo nitrile intermediate **26**, which was successfully reacted with guanidine to afford 6-[(2,5-dimethoxyphenyl)methyl]-5-methylpyrido[2,3-*d*]pyrimidine-2-amine (**28**) (Scheme 5) [21].

**Scheme 6:**
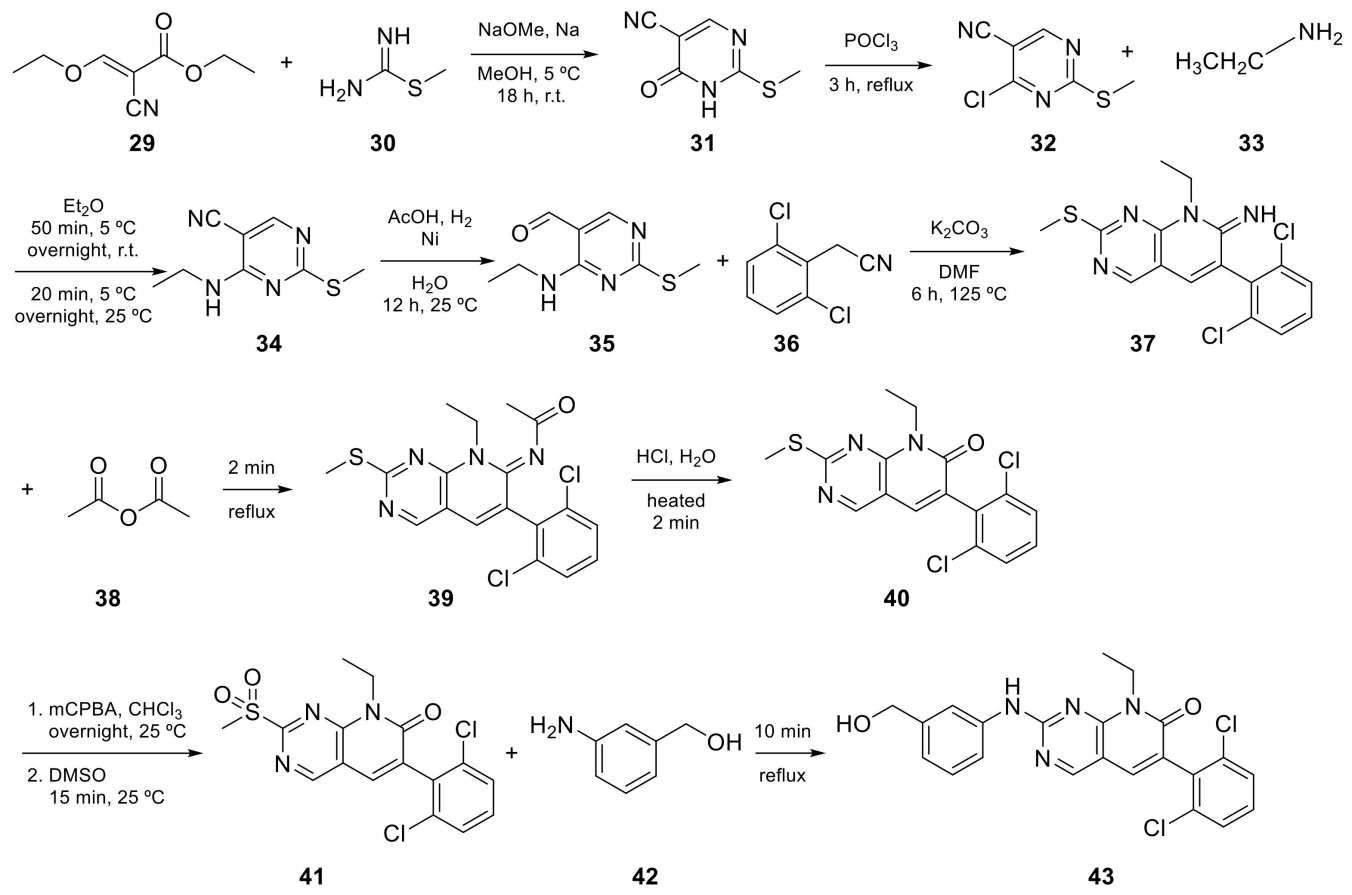
Synthesis of 6-(2,6-dichlorophenyl)-2-([3-(hydroxymethyl)phenyl]amino)-8-ethyl-7*H*,8*H*-pyrido[2,3-*d*]pyrimidin-7-one (**43**) [25].

A.M. Doherty et al. [25] previously reported the identification of a lead, 2-amino-6-(2,6-dichlorophenyl)-8-methyl-8*H*-pyrido[2,3-*d*]pyrimidin-7-one, as a good inhibitor of PDGFr (platelet-derived growth factor) or FGFr (fibroblast growth factor) tyrosine kinase [26]. Tyrosine kinases are enzymes that catalyze a specific phosphorylation of tyrosine residues on proteins. These enzymes are implicated in diverse mechanisms in cell life. Finding selective inhibitors of such enzymes may make it possible to fight against angiogenesis, restenosis atherosclerosesis and tumor growth. A library of compounds was synthesized and tested. The synthetic pathway used to obtain these interesting core structures was applied to the 6-(2,6-dichlorophenyl)-2-{[3-(hydroxymethyl)phenyl]amino}-8-ethyl-7*H*,8*H*-pyrido[2,3-*d*]pyrimidin-7-one (**43**) and is depicted in Scheme 6 (Table 1, entry 6). Biological tests carried out on the library of compounds obtained showed that the activity of derivatives bearing an ethyl group on N8 was four-fold better than for N8-methylated analogues.

**Scheme 7:**
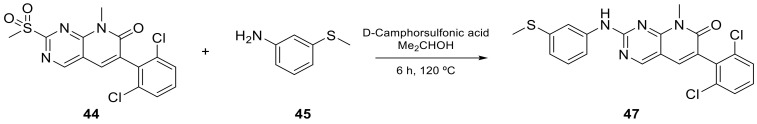
Synthesis of 6-(2,6-dichlorophenyl)-8-methyl-2-([3-(methylsulfanyl)phenyl]amino)-7*H*,8*H*-pyrido [2,3-*d*]pyrimidin-7-one (**47**), PD-173955 [17].

PD-173955 (6-(2,6-dichlorophenyl)-8-methyl-2-([3-(methylsulfanyl)phenyl]amino)-7*H*,8*H*-pyrido [2,3-*d*]pyrimidin-7-one (**47**) (Scheme 7, Table 1, entry 7) is another example of this family of pyrido[2,3-*d*]pyrimidines. It is known to target the ephrin receptor (EPH) family of proteins that are overexpressed in some cancers [17]. This compound, **47**, was easily obtained from previously synthesized starting material by a reaction with 3-methylthio-aniline in the presence of camphorsulfonic acid in isopropylalcohol.

Mitogen-activated protein kinase 14 (also known as p38-α) is an enzyme encoded by the MAPK14 gene in humans. P38 mitogen-activated protein kinase is a type of mitogen-activated protein kinase (MAPK) that can respond to stress stimuli (such as cytokines, ultraviolet radiation, heat shock and osmotic shock), and is involved in cell differentiation, apoptosis and autophagy. Recent data have shown that the p38 stress MAPK pathway may regulate Ras-dependent and independent proliferation, transformation, invasion and cell death through isoform-specific mechanisms, thereby playing a role in suppressing cancer [27,28].

**Scheme 8:**
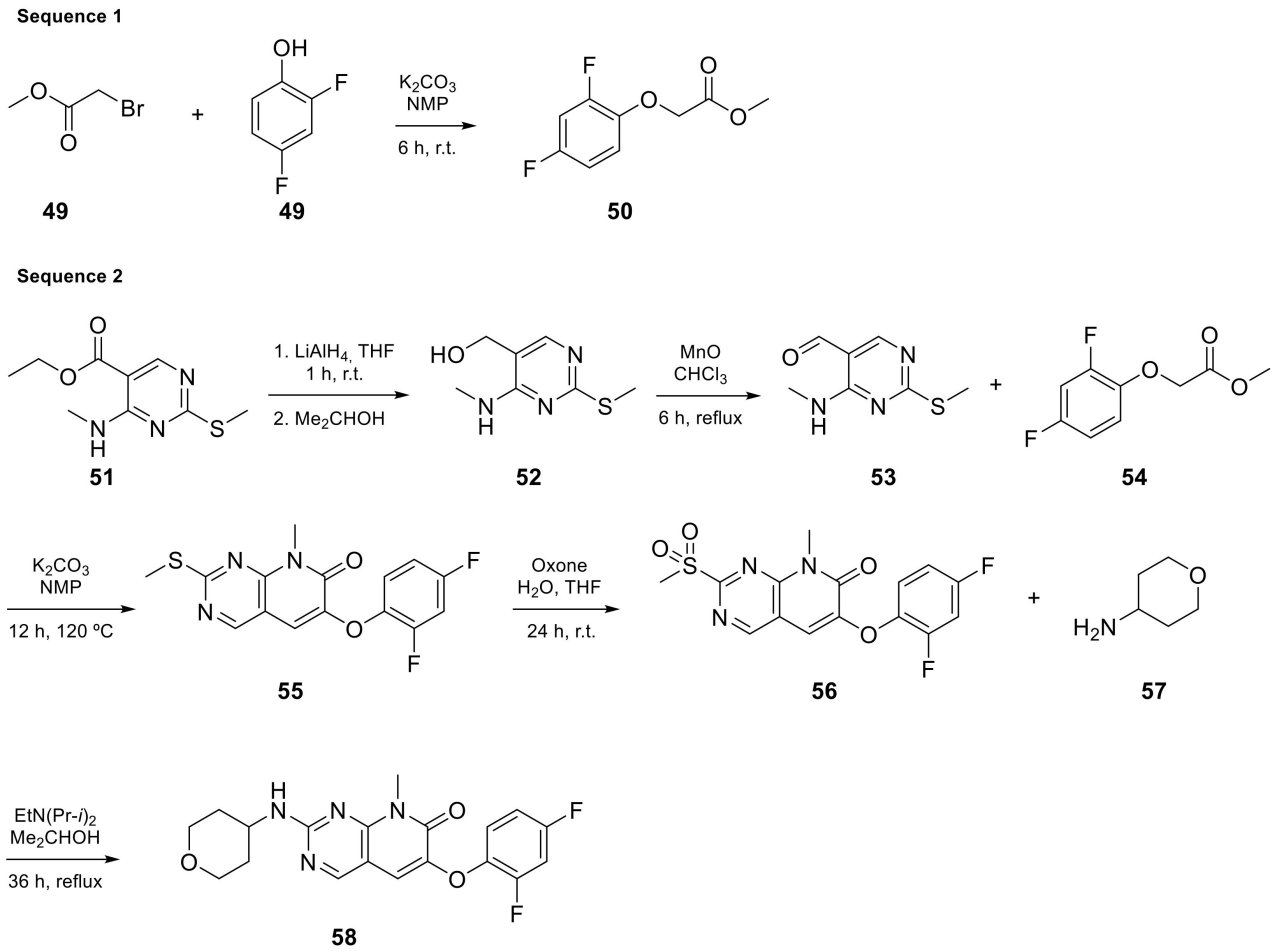
Synthesis of 6-(2,4-difluorophenoxy)-8-methyl-2-[(oxan-4-yl)amino]-7*H*,8*H*-pyrido[2,3-*d*]pyrimidin-7-one (**58**) [29].

The synthesis of kinase inhibitors such as 6-(2,4-difluorophenoxy)-8-methyl-2-[(oxan-4-yl)amino]-7*H*,8*H*-pyrido[2,3-*d*]pyrimidin-7-one (**58**) (Scheme 8, Table 1, entry 8) was performed via the following 6 step synthetic pathway using substituted 2,4-difluorophenol obtained beforehand, according to sequence 1 [29].

**Scheme 9:**
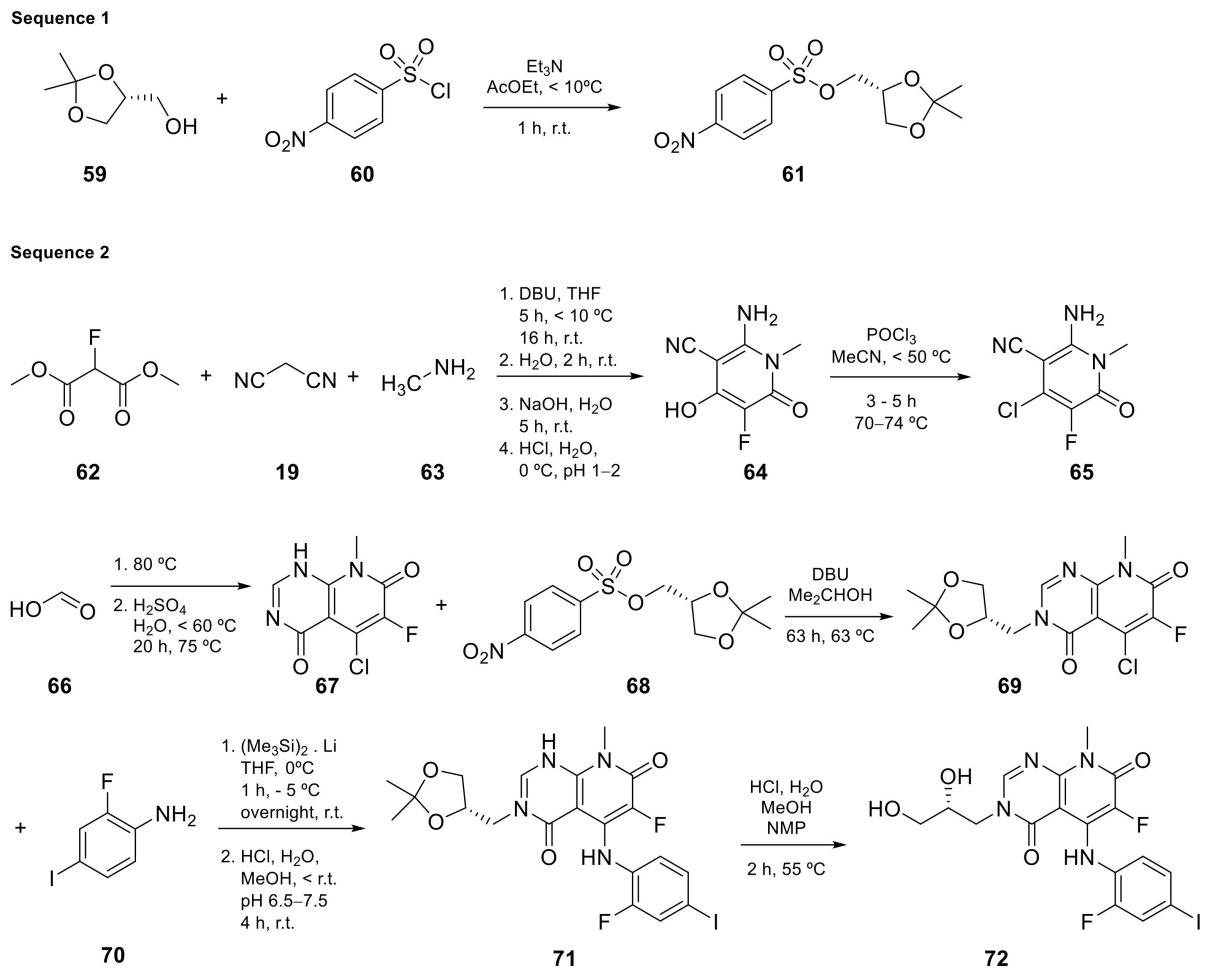
Synthesis of 3-[(2*R*)-2,3-dihydroxypropyl]-6-fluoro-5-[(2-fluoro-4-iodophenyl)amino]-8-methyl-3*H*,4*H*,7*H*,8*H*-pyrido[2,3-*d*]pyrimidine-4,7-dione (**72**) [33].

TAK-733 (Scheme 9, Table 1, entry 9) exhibits potent enzymatic and cell activity against a constitutively active MEK enzyme and against ERK phosphorylation in cells. TAK-733 demonstrates broad activity in most melanoma cell lines and has been used to study advanced metastatic melanoma and advanced non-hematological malignancies [30,31,32].

Li et al. [33] reported the development of an efficient approach to achieve TAK-733 using fewer steps and with higher yields. A polysubstituted fluoropyridone **64** was produced in one pot via a three-step cascade reaction: condensation between α-fluoromalonate and malononitrile, methyl amide formation, and intramolecular cyclization. Hydroxyl functionality chlorination and cyclization with formic acid afforded the pyridopyrimidone **67**. Ensuing *N*-alkylation with the nosylate of (R)-glycerol acetonide and chlorine displacement with 2-fluoro-4-iodoaniline was successfully achieved. The final step consisted of the acid-catalyzed deprotection of the acetonide functionality to afford the pyrido[2,3-*d*]pyrimidine-4,7-dione **72** (Scheme 9) [33].

**Scheme 10:**
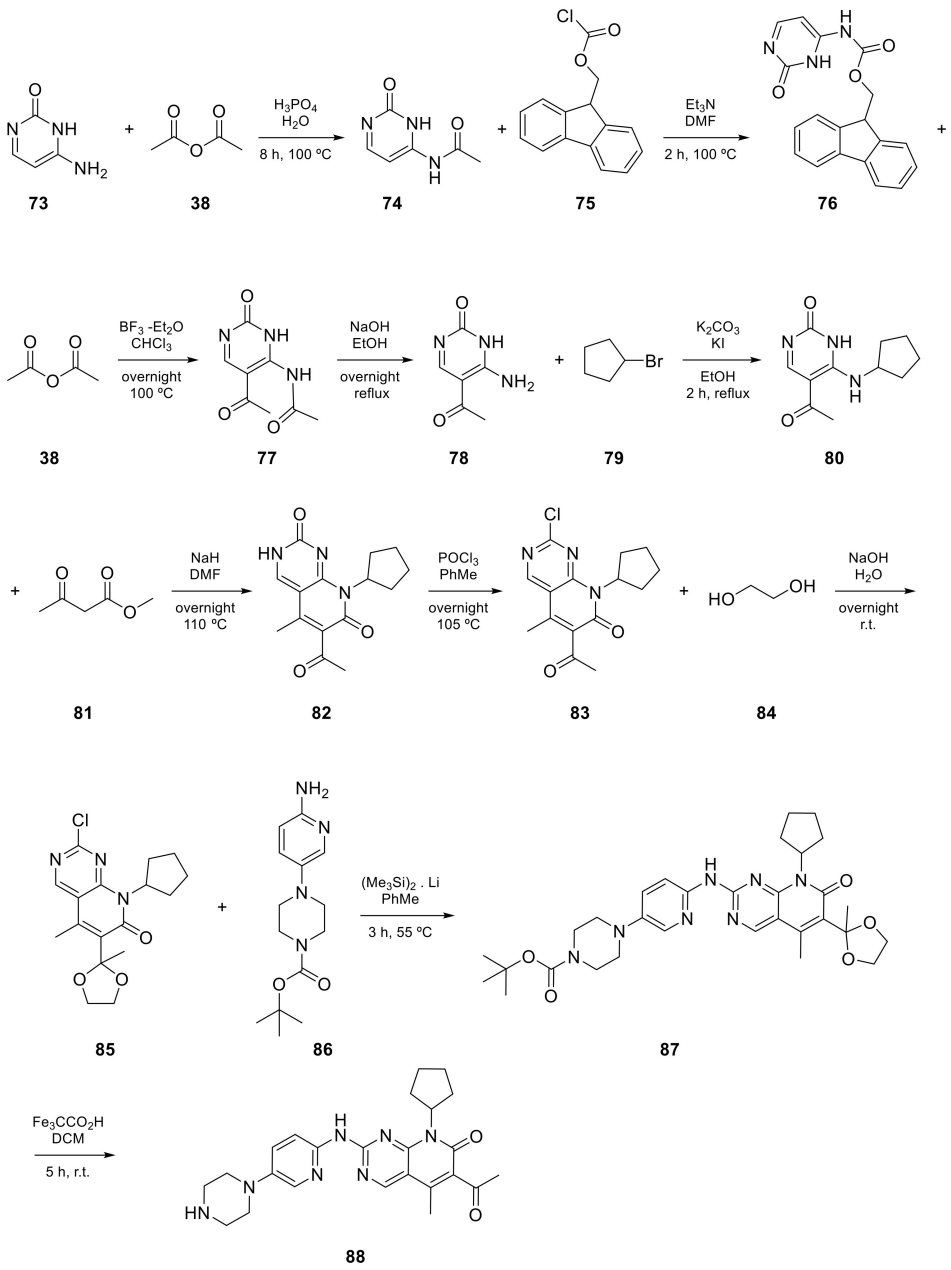
Synthesis of 6-acetyl-8-cyclopentyl-5-methyl-2-([5-(piperazin-1-yl)pyridin-2-yl]amino)-7*H*,8*H*-pyrido[2,3-*d*]pyrimidin-7-one (**88**), Palbociclib [38].

Palbociclib (**88** in Scheme 10, Table 1, entry 10) is a breast cancer drug developed by Pfizer. This is a piperazine pyridopyrimidine that plays a role in the cell cycle mechanism. Pyridopyrimidine referenced as a second-generation cyclin-dependent kinase inhibitor. It can be used as an endocrine-based chemotherapy drug in combination with other anti-tumor drugs to treat HER2-negative and HR-positive advanced or metastatic breast cancer.

It was in the context of these treatments that Palbociclib was initially approved by the FDA in March 2015. The results of post-marketing reports and electronic health records, updated in April 2019, show its safety and clinical efficacy [34,35]. The target of Palbociclib is Cyclin-dependent kinase 4 and Cyclin-dependent kinase 6. Cyclin-dependent kinase (CDK) is a protein kinase characterized by the need for a separate subunit—cyclin—to provide a domain necessary for enzymatic activity. CDK plays an important role in the control of cell division and regulates transcription in response to a variety of extracellular and intracellular signals. The CDK inhibitor protein is a protein that inhibits CDK. Some of them act as tumor suppressor proteins. Cell cycle progression is delayed or stopped by cyclin-dependent kinase inhibitors. CDK4/6 inhibitors are a class of drugs that target specific enzymes, called CDK4 and CDK6. CDK4/6 inhibitors disrupt the signals that stimulate the proliferation of malignant (cancer) cells [36,37]. The synthesis of Palbociclib was reported by Chu et al. in 2020, as described in Scheme 10 [38].

**Scheme 11:**
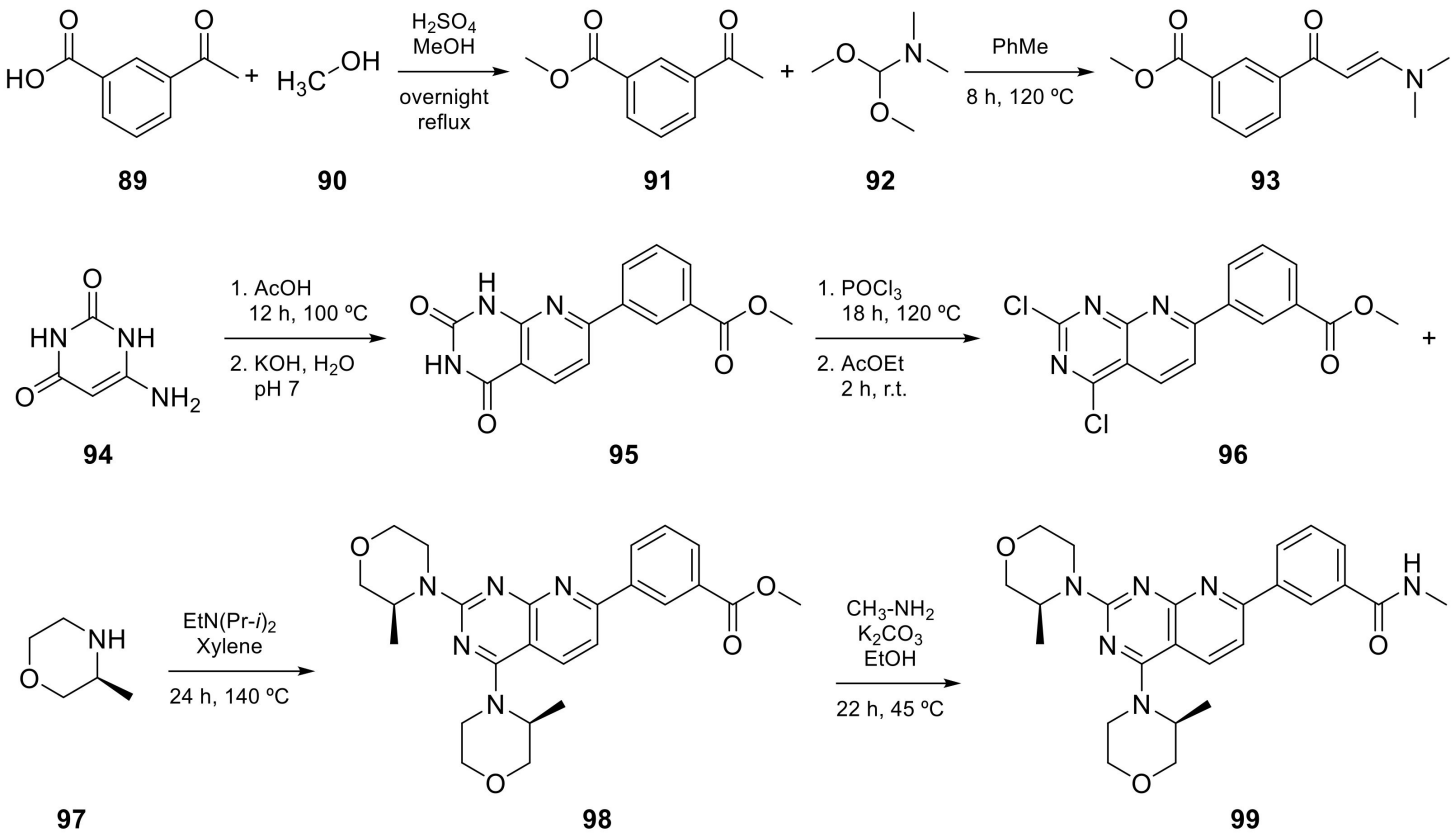
Synthesis of 3-(2,4-bis[(3*S*)-3-methylmorpholin-4-yl]pyrido[2,3-*d*]pyrimidin-7-yl)-*N*-methylbenzamide (**99**) [40].

Vistusertib (**99** in Scheme 11, Table 1, entry 11) is being studied for the treatment of advanced gastric adenocarcinoma [39]. Vistusertib or AZD2014 is a novel mTOR inhibitor. Meng et al. [40] reported a synthesis of Vistusertib in 2019 from the 3-acetylbenzoic acid, which was transformed to methyl ester **91**, and then treated with dimethylformamide dimethylacetal to furnish the enaminone **93**. The enaminone heteroannulation reaction with 6-aminouracil was accomplished in acidic conditions to allow pyrido[2,3-*d*]pyrimidine **95**. This compound was chlorinated with phosphorus oxychloride to obtain 2,4-dichloropyrido[2,3-*d*]pyrimidine **96**. In the next stage, this team showed an improvement when they realized that xylene was the ideal solvent for the substitution reaction. In this way, Vistusertib was synthesized in one step. When potassium carbonate was used as the reaction accelerator, the intermediate was subjected to aminolysis in a 30% methylamine alcohol solution to obtain the final product **99** [40].

**Scheme 12:**
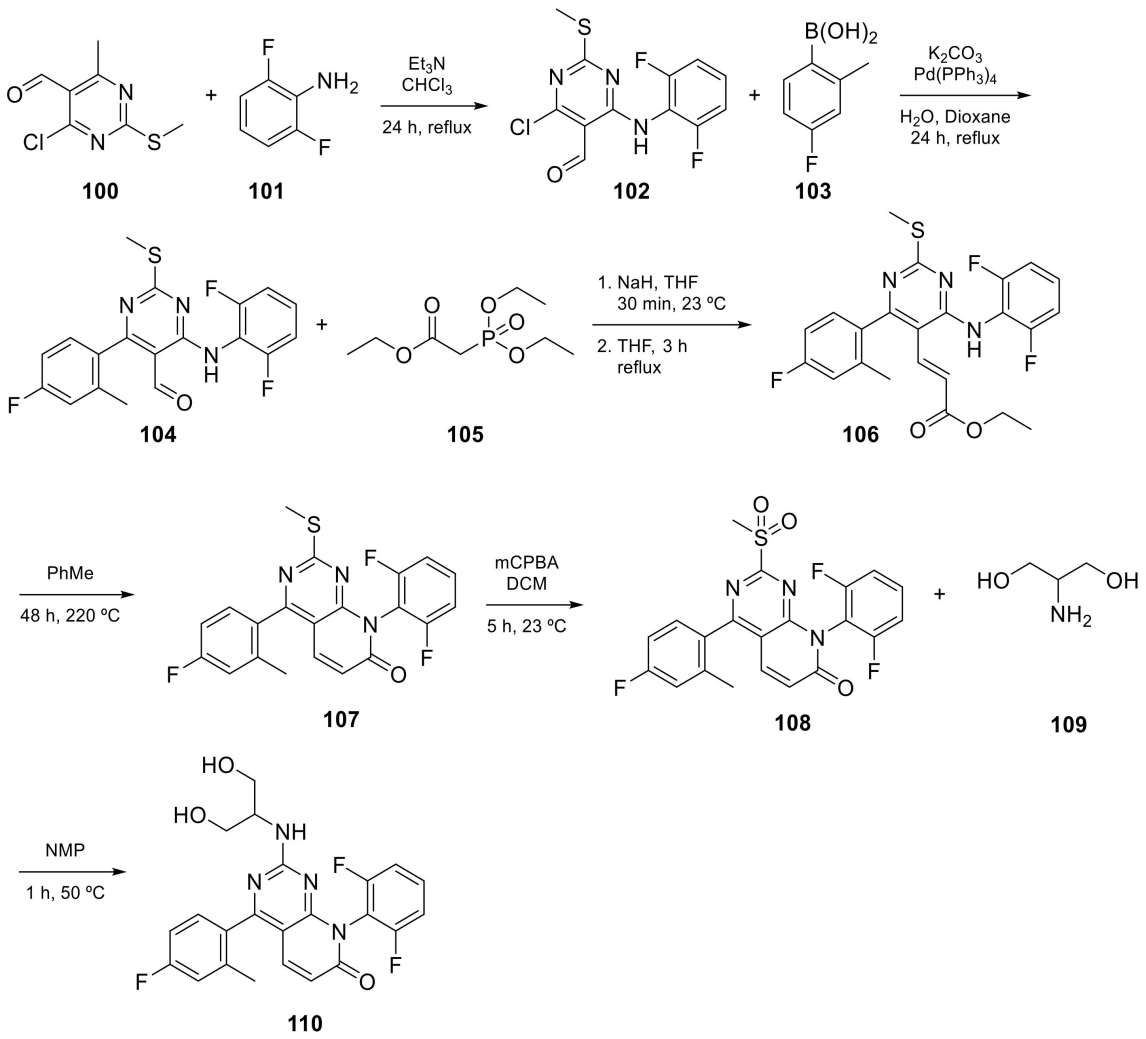
Synthesis of 8-(2,6-difluorophenyl)-2-[(1,3-dihydroxypropan-2-yl)amino]-4-(4-fluoro-2-methylphenyl)-7*H*,8*H*-pyrido[2,3-*d*]pyrimidin-7-one, Dilmapimod (**110**) [47].

Dilmapimod (**110** in Scheme 12, Table 1, entry 12) has been used to study the treatment and diagnostic tests of nerve trauma, inflammation, pain, neuropathy, arthritis, rheumatoid disease and coronary heart disease. Dilmapimod (SB-681323) is a p38 MAP kinase inhibitor with potential use in inflammatory diseases such as RA (rheumatoid arthritis). Previous p38 MAP kinase inhibitors were hampered by hepatotoxicity during development [41,42]. The target of Dilmapimod is the Tumor necrosis factor/Interleukin-1 beta/Interleukin-6.

The Tumor Necrosis Factor (TNF) belongs to a family of numerous transmembrane proteins with a homologous TNF domain involved, in particular, in the inflammatory cascade and in other important biological events. As a pro-inflammatory cytokine, TNF may be implicated in various inflammation-related cancers. The most active tumor necrosis factor inhibitors (anti-TNF drugs) are monoclonal antibodies against TNF-α (TNFα). As the name suggests, TNF activates the immune system help to kill cancer cells. Recombinant TNF, administered by isolated limb perfusion, is a potent cause of endothelial cell death and subsequent tumor necrosis. TNF inhibitors are antibodies made from human or animal tissues in the laboratory. Once they are placed in blood, they cause a reaction in the immune system which prevents inflammation [43,44].

Interleukins regulate cell growth, differentiation and movement. They are especially important in stimulating immune responses (such as inflammation). Interleukin-1β is a form of interleukin 1, which is mainly made by one type of white blood cells (macrophages) and helps another type of white blood cells (lymphocytes) fight infection. Generally, interleukin 1 is responsible for the production of inflammation and the promotion of fever and sepsis. IL-1α inhibitors are under development to interrupt these processes and treat diseases. IL-6 is responsible for stimulating the synthesis of acute phase protein and the production of neutrophils in the bone marrow. It supports the growth of B cells and antagonizes regulatory T cells [45,46].

**Scheme 13:**
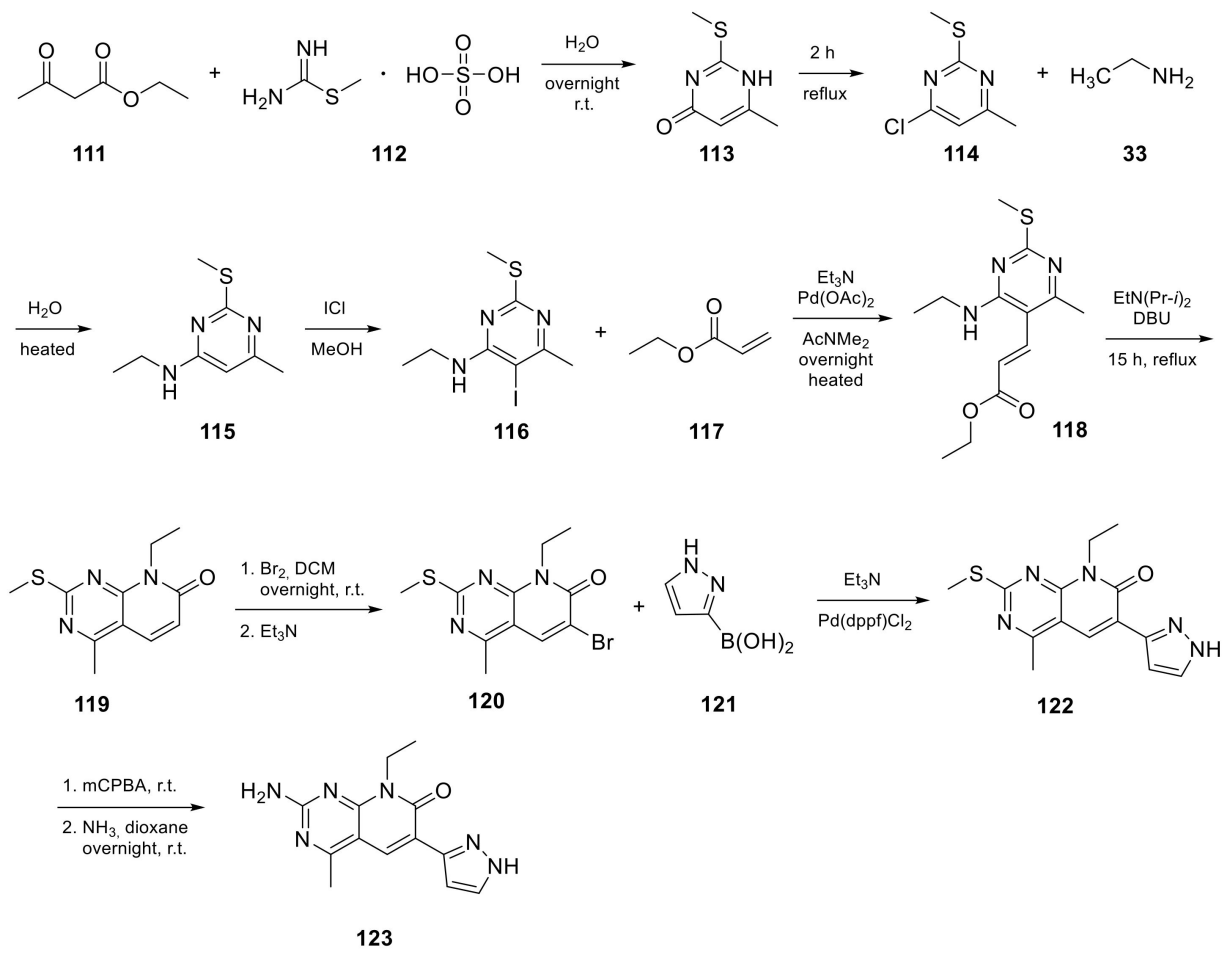
Synthesis of 2-amino-8-ethyl-4-methyl-6-(1*H*-pyrazol-5-yl)-7*H*,8*H*-pyrido[2,3-*d*]pyrimidin-7-one, Voxtalisib (**123**) [49].

Voxtalisib (**113** in Scheme 13, Table 1, entry 13) has been used in research treatment trials for cancer, melanoma, lymphoma, glioblastoma and breast cancer [48]. This drug acts as an inhhibitor of two targets, the kinase enzymes phosphatidylinositol-3-kinase PI3K and rapamycin mTOR. It could be used for the treatment of various types of cancer.

Romanelli et al. [49] patented, in 2014, the synthesis of Voxtalisib following the synthetic pathway described in Scheme 13.

**Scheme 14:**
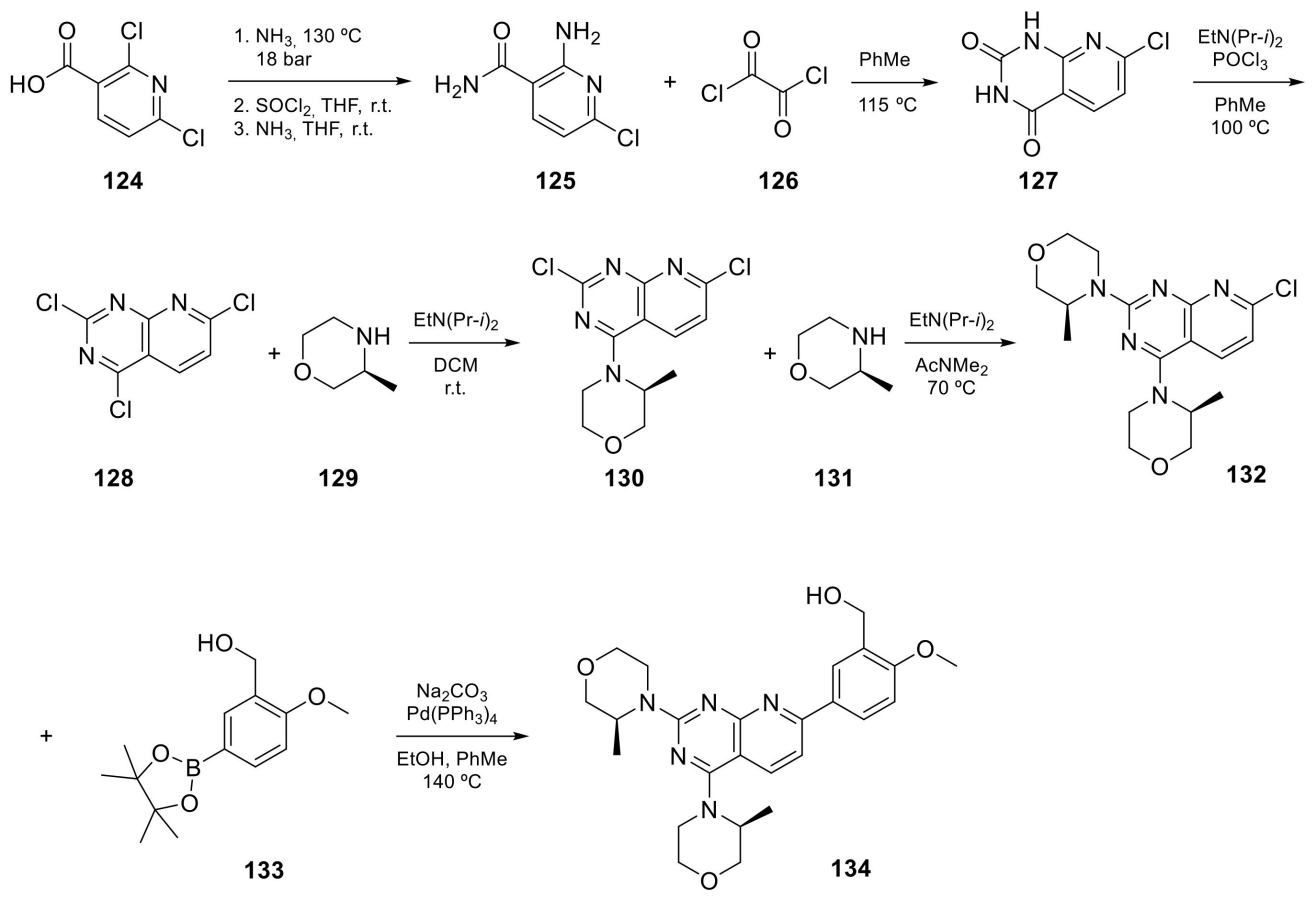
Synthesis of (5-{2,4-bis[(3*S*)-3-methylmorpholin-4-yl]pyrido[2,3-*d*]pyrimidin-7-yl}-2-methoxyphenyl)methanol (**134**), AZD8055 [51].

AZD8055 (**134** in Scheme 14, Table 1, entry 14) has been used in treatment trials of cancer, lymphoma, solid tumors, malignant glioma and brainstem glioma [50]. AZD8055 is a selective ATP-competitive mTOR kinase inhibitor and inhibits cell proliferation and Pass et al. described its synthesis. 3*S*-Methylmorpholine was added successively at the C4 and then C2 positions of the trichloro intermediate **128** to give the chlorinated pyrido[2,3-*d*]pyrimidine **132** which was then reacted with a boronic ester using palladium coupling to achieve the final compound [51].

**Scheme 15:**
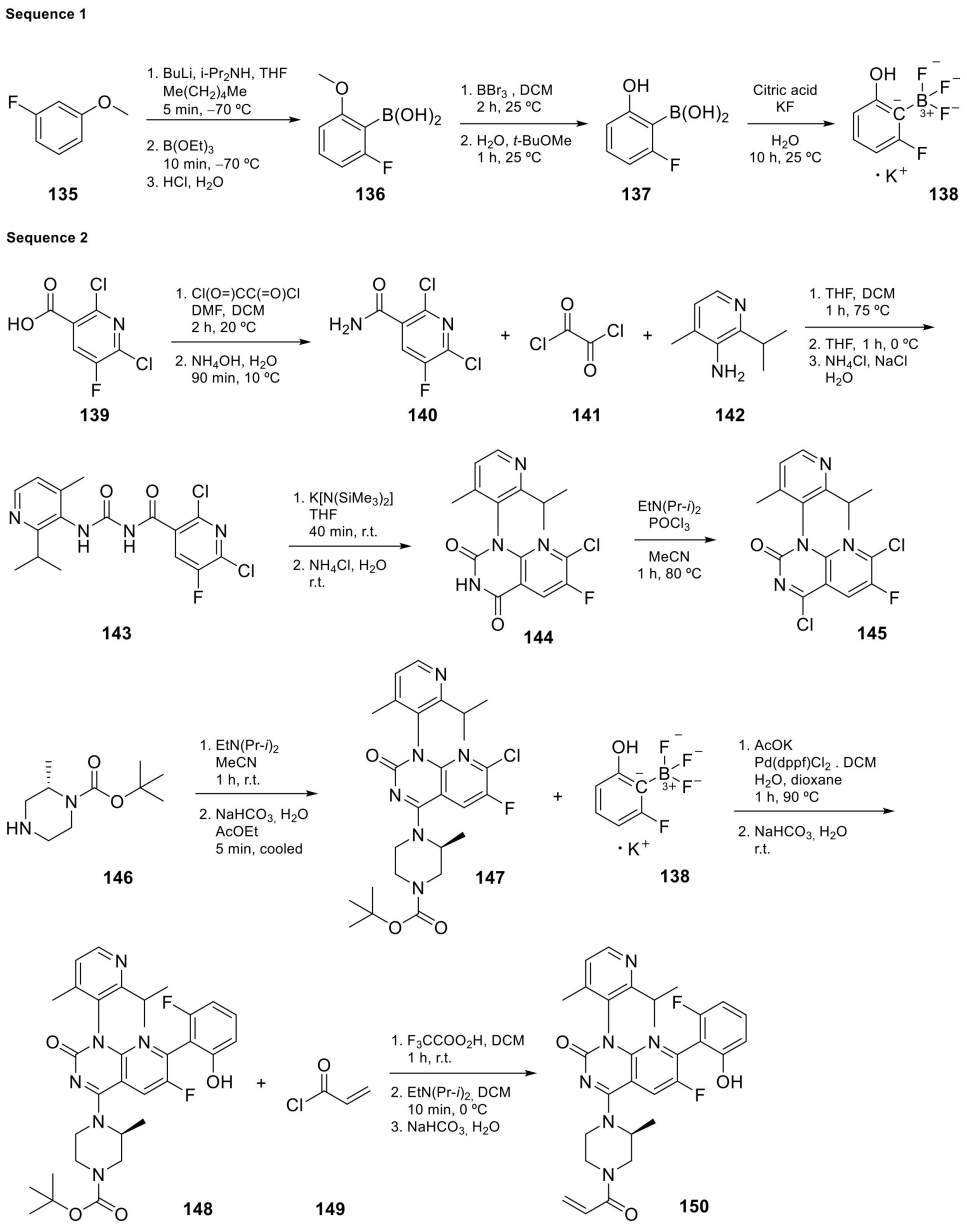
6-fluoro-7-(2-fluoro-6-hydroxyphenyl)-1-[4-methyl-2-(propan-2-yl)pyridin-3-yl]-4-[(2*S*)-2-methyl-4-(prop-2-enoyl)piperazin-1-yl]-1*H*,2*H*-pyrido[2,3-*d*]pyrimidin-2-one (**150**), AMG-510 [56].

AMG-510 (**150** in Scheme 15, Table 1, entry 15) is an experimental KRAS inhibitor that is under study for the treatment of KRAS G12C mutant non-small cell lung cancer, colorectal cancer and appendix cancer. AMG-510 is a KRAS inhibitor derived from acrylamide and developed by Amgen. It is currently undergoing clinical trials for solid tumors with KRAS G12C mutations. This mutation accounts for more than 50% of all KRAS mutations. It is the first experimental KRAS inhibitor [52,53]. The target is the GTPase KRas.

Like other members of the ras subfamily, the KRAS protein is a GTPase and an early participant in many signal transduction pathways. Due to the presence of an isoprene group at the C-terminus, KRAS is usually bound to the cell membrane. The KRAS gene provides instructions for the preparation of a protein called K-Ras, which is part of a signaling pathway called the RAS/MAPK pathway. This protein transmits signals from outside the cell to the nucleus. One of the most common mutations is KRAS G12C, which occurs in approximately 13% of NSCLC and 3–5% of CRC. Due to its unusual shape, mutant KRAS has long been known as a non-drug target. Compared with other proteins, the relatively smooth protein structure means that it is difficult to design inhibitors that bind to surface grooves, which has hindered the progress of drug development for many years [54,55].

In 2020, Parsons et al. [56] patented their improvements in the synthesis of key intermediates of the AMG-510 KRAS G12C inhibitor, as described in Scheme 15. The synthesis is carried out in fifteen steps, one of the reagents having to be obtained beforehand according to sequence 1.

**Scheme 16:**
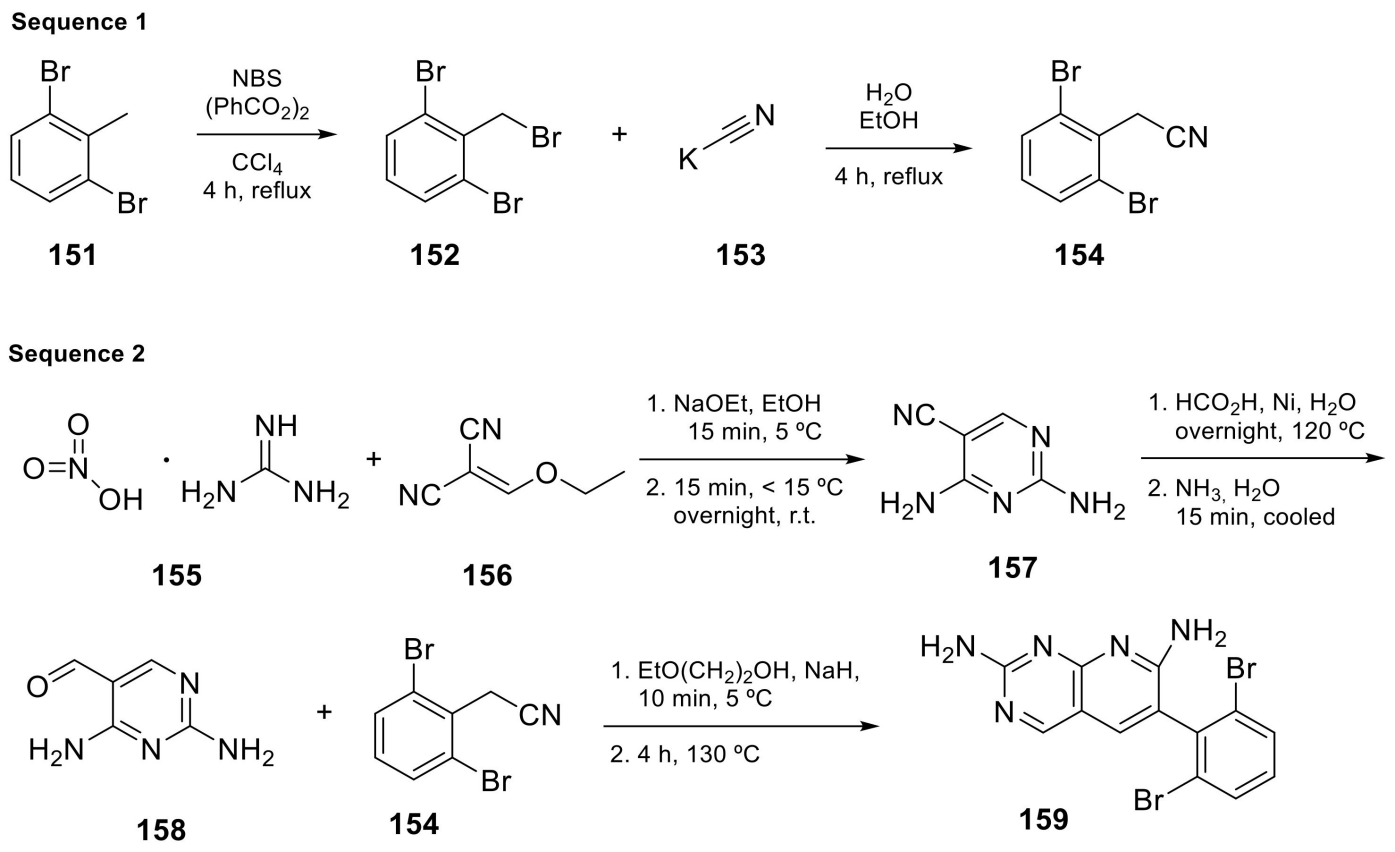
Synthesis of 6-(2,6-dibromophenyl)pyrido[2,3-*d*]pyrimidine-2,7-diamine (**159**) [57].

Zega et al. reported in 2012 the synthesis of 6-(2,6-dibromophenyl)pyrido[2,3-*d*]pyrimidine-2,7-diamine (**159**) (Scheme 16, Table 1, entry 16) which has biotin carboxylase as a biological target. They obtained 2,4-diamino-5-cyanopyrimidine **157** by condensation of guanidine nitrate with ethoxymethylenemalononitrile (**156**) in ethanol and sodium ethoxide, to achieve the free guanidine base. A Raney nickel catalyst in 98% formic acid as a solvent allowed the reduction of the cyano group. Then 2,4-diaminopyrimidine-5-carboxaldehyde **158** was condensed with benzylnitrile under basic conditions to yield 2,7-diaminopyridopyrimidine **159** (Scheme 16) [57].

**Scheme 17:**
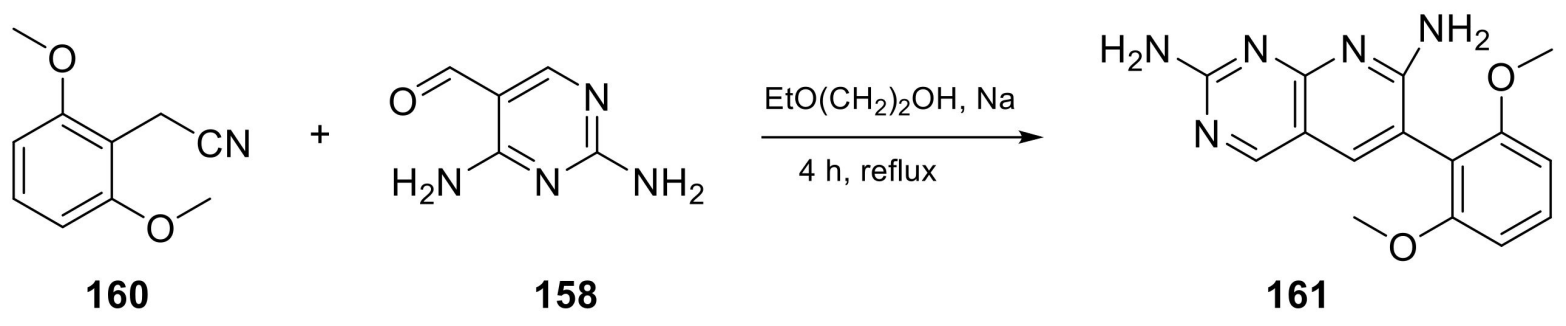
Synthesis of 6-(2,6-dimethoxyphenyl)pyrido[2,3-*d*]pyrimidine-2,7-diamine (**161**) [25,58].

Using the same protocol, the analogous 6-(2,6-dimethoxyphenyl)pyrido[2,3-*d*]pyrimidine-2,7-diamine (**161**) (Scheme 17, Table 1, entry 17) was efficiently synthesized, targeting the same biotin carboxylase [25,58].

**Scheme 18:**
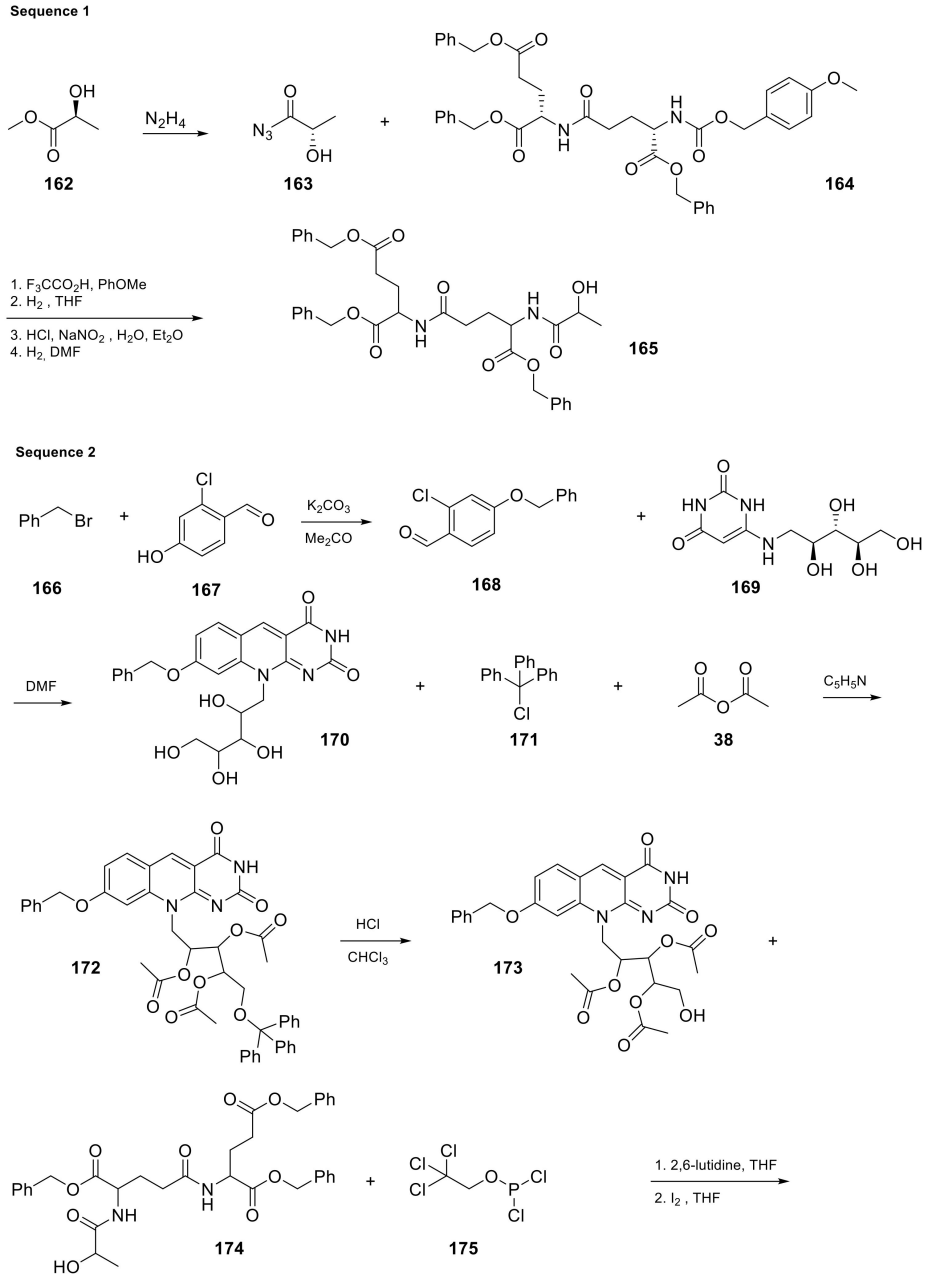

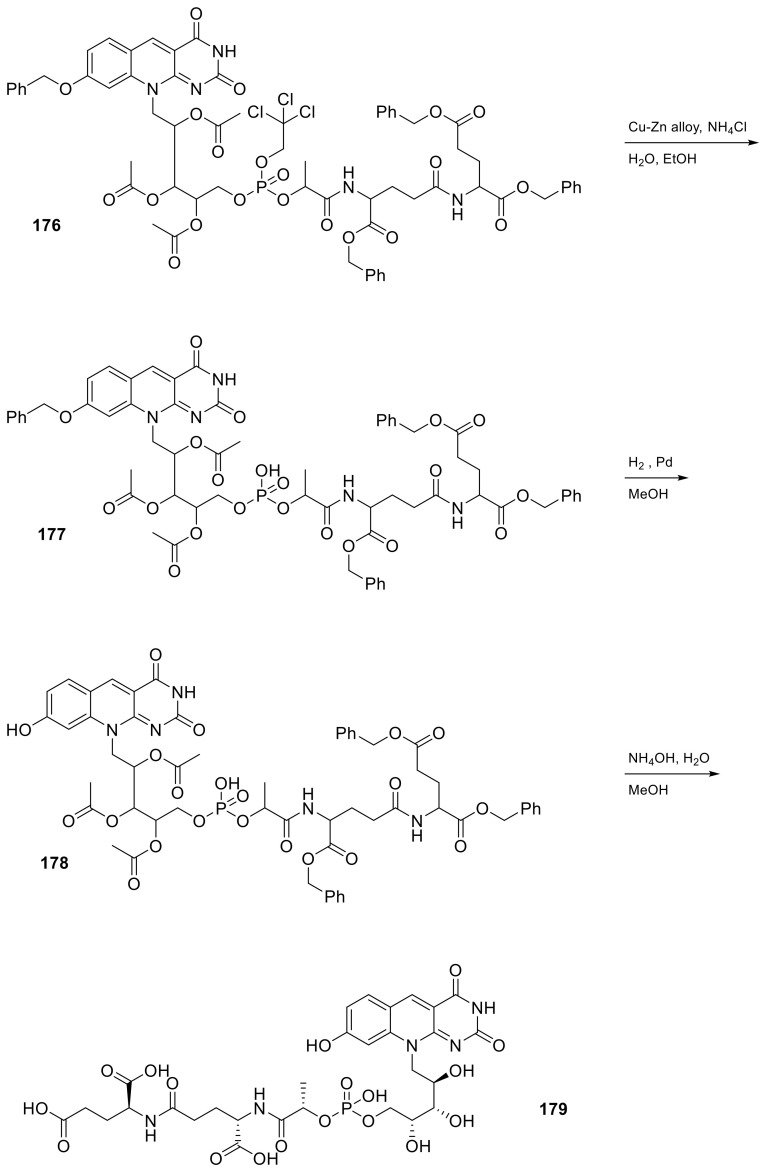
(2*S*)-2-[(4*S*)-4-carboxy-4-[(2*S*)-2-([hydroxy(([(2*R*,3*S*,4*S*)-2,3,4-trihydroxy-5-(8-hydroxy-2,4-dioxo-2*H*,3*H*,4*H*,10*H*-pyrimido[4,5-*b*]quinolin-1-yl)pentyl]oxy))phosphoryl]oxy)propanamido]butanamido]pentanedioic acid (**179**) [59].

Yoneda et al. reported in 1990 the first total synthesis of the coenzyme factor 420, the (2*S*)-2-[(4*S*)-4-carboxy-4-[(2*S*)-2-([hydroxy(([(2*R*,3*S*,4*S*)-2,3,4-trihydroxy-5-(8-hydroxy-2,4-dioxo-2*H*,3*H*,4*H*,10*H*-pyrimido[4,5-*b*]quinolin-1-yl)pentyl]oxy))phosphoryl]oxy)propanamido]butanamido]pentanedioic acid (**179** in Scheme 18, Table 1, entry 18). This team’s approach consisted of using two routes without the need to protect functional groups. The desired product was attained by the creation of a phosphotriester bond concerning a protected 8-hydroxy-10-*o*-ribityl-5-deazaisoalloxazine moiety and a peptide moiety, (l-lactoyl-y-l-glutamyl)-l-glutamic acid tribenzyl ester, by the phosphite triester method using 2,2,2-trichloroethyl phosphorodichloridite, followed by consecutive deprotection methods (Scheme 18) [59].

### 2.2. Pyrido[3,4-d]pyrimidine

**Scheme 19:**
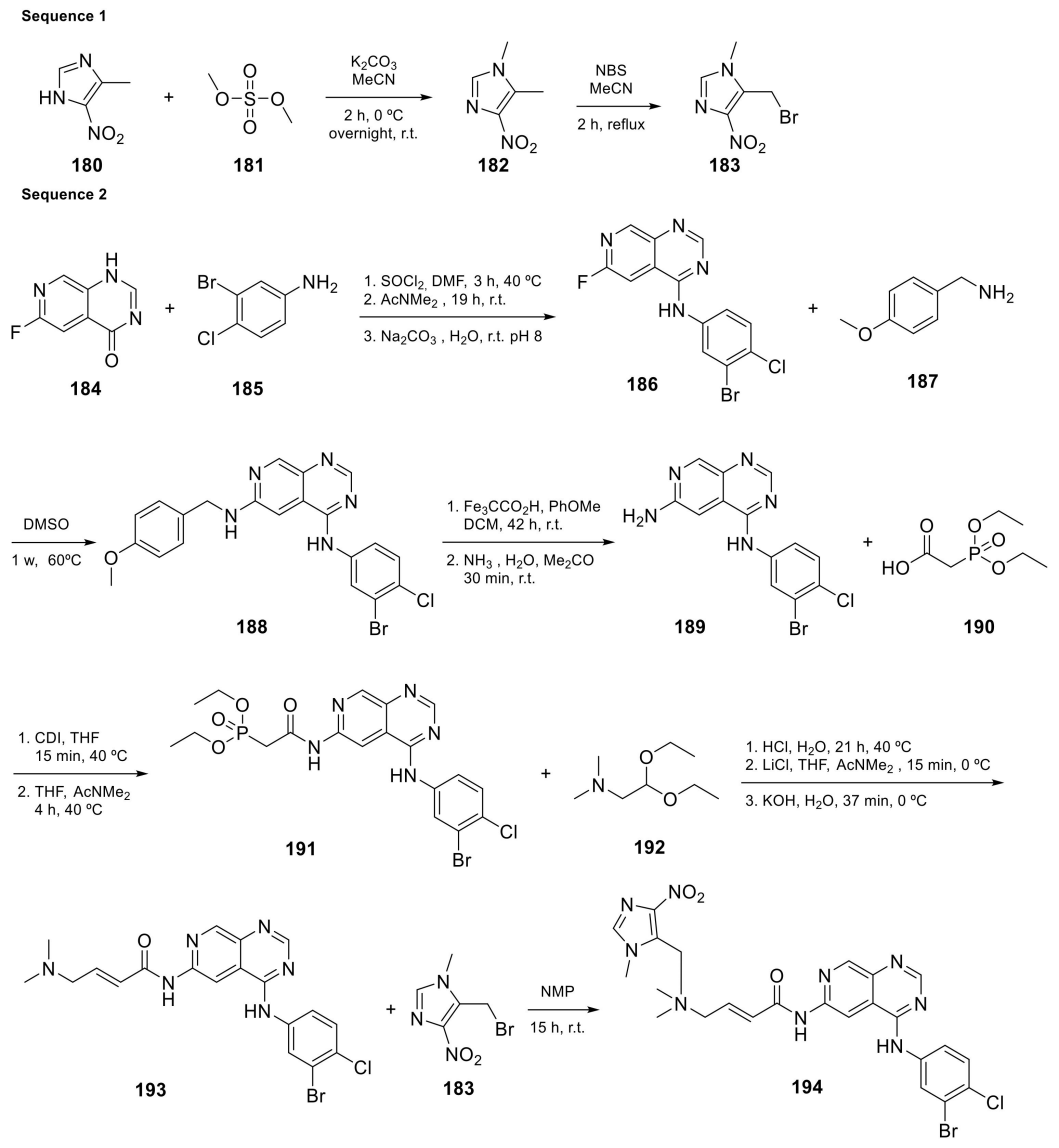
[(2*E*)-3-({4-[(3-bromo-4-chlorophenyl)amino]pyrido[3,4-*d*]pyrimidin-6-yl}carbamoyl)prop-2-en-1-yl]dimethyl[(1-methyl-4-nitro-1*H*-imidazol-5-yl)methyl]azanium (**194**) [61].

This class of pyridopyrimidine is mainly referenced with kinase activity. The first example mentioned herein is Tarloxotinib (**194** in Scheme 19). It is being studied in the clinical trial NCT03743350 (NSCLC exon 20 or HER2 activating mutation) [60]. This molecule is a kinase inhibitor targeting all members of the HER family, with a novel mechanism of action. It is a hypoxia-activated prodrug that releases an active metabolite irreversibly targeting the kinase. The goal is to inhibit only HER kinases in tumor cells. Tarloxotinib is a Pan-HER kinase inhibitor.

Carlin et al. [61] patented in 2015 the preparation of 4-anilinopyrido[3,4-d]pyrimidine prodrugs (Scheme 19, Table 1, entry 19) as kinase inhibitors useful for cancer treatment. The procedure is described in Scheme 19 with classical synthetic methodologies affording the expected compound **194** in twelve steps.

**Scheme 20:**
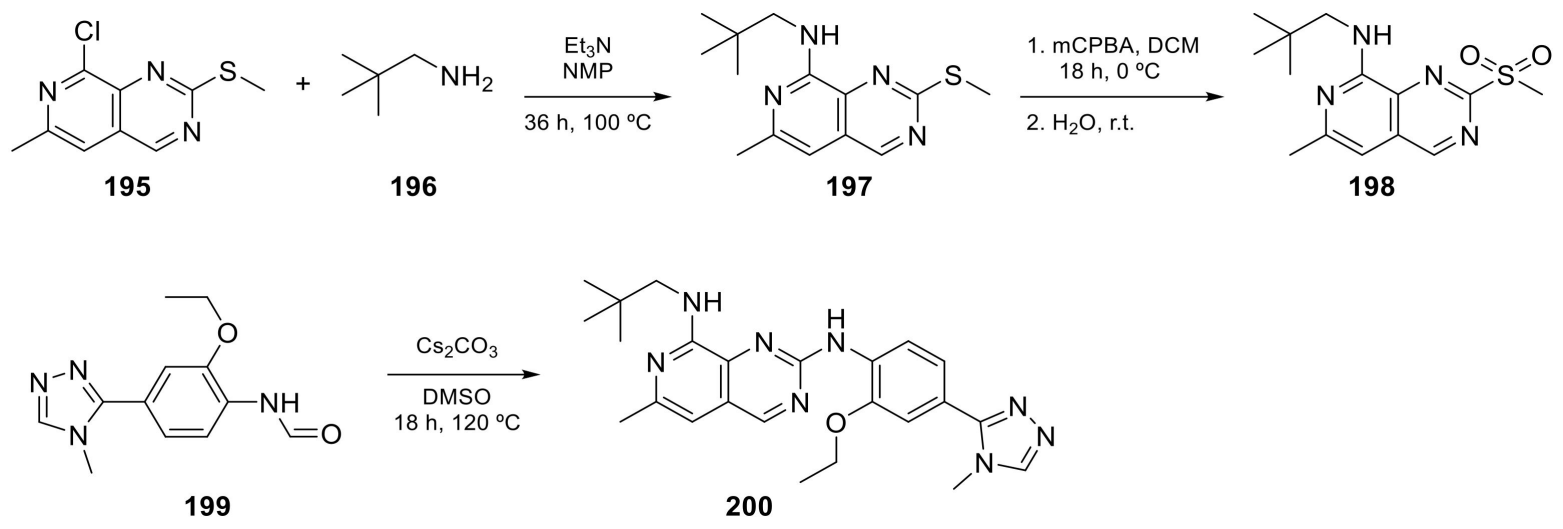
*N*8-(2,2-dimethylpropyl)-*N*2-[2-ethoxy-4-(4-methyl-4*H*-1,2,4-triazol-3-yl)phenyl]-6-methylpyrido[3,4-*d*]pyrimidine-2,8-diamine (**200**), BOS172722 [62].

The second example is the BOS172722 derivative (**200** in Scheme 20, Table 1, entry 20). This compound, in combination with paclitaxel, was tested in vivo for the treatment of triple hormone receptor-negative breast cancer demonstrating a promising synergy. This selective monopolar spindle 1 (Mps1) kinase inhibitor has been identified as a potential anti-cancer agent because it is involved in the division of cancer cells. This is, therefore, an attractive target for cancer therapy [62,63]. It has the dual specificity protein kinase TTK as the target.

Monopolar spindle 1 (Mps1/TTK) is a conserved serine/threonine kinase from yeast to humans. It has been shown to be a key kinase that activates the spindle assembly checkpoint (SAC) to ensure the proper distribution of chromosomes to progeny cells. It is also one of the main components of SAC, which can ensure that the cells do not develop from mid-term to late-term until the boom is properly connected to the microtubules and proper tension is applied to the mid-term plate. Cancer cells rely heavily on MPS1 to cope with the abnormal number of chromosomes in aneuploidy caused by MPS1. It has been found that this kinase is upregulated in many types of tumors. Mps1 is an attractive oncology target because of its high expression level in cancer cells and the correlation between its expression level and the histological grade of the cancer. Based on the kinase profile, the compounds selectively inhibit MPS1 and reduce the phosphorylation of MPS1 and Phospho-HH3 signaling, successfully [64,65].

Hoelder et al. reported the synthesis of **200** (Scheme 20, Table 1, entry 20) in 2018. The chloride displacement allowed the addition of the amine and oxidation with m-CPBA gave the sulfone. The sulfone displacement with formamide using Cs_2_CO_3_/DMSO gave the final product **200** [62].

### 2.3. Pyrido[4,3-d]pyrimidine

**Scheme 21:**
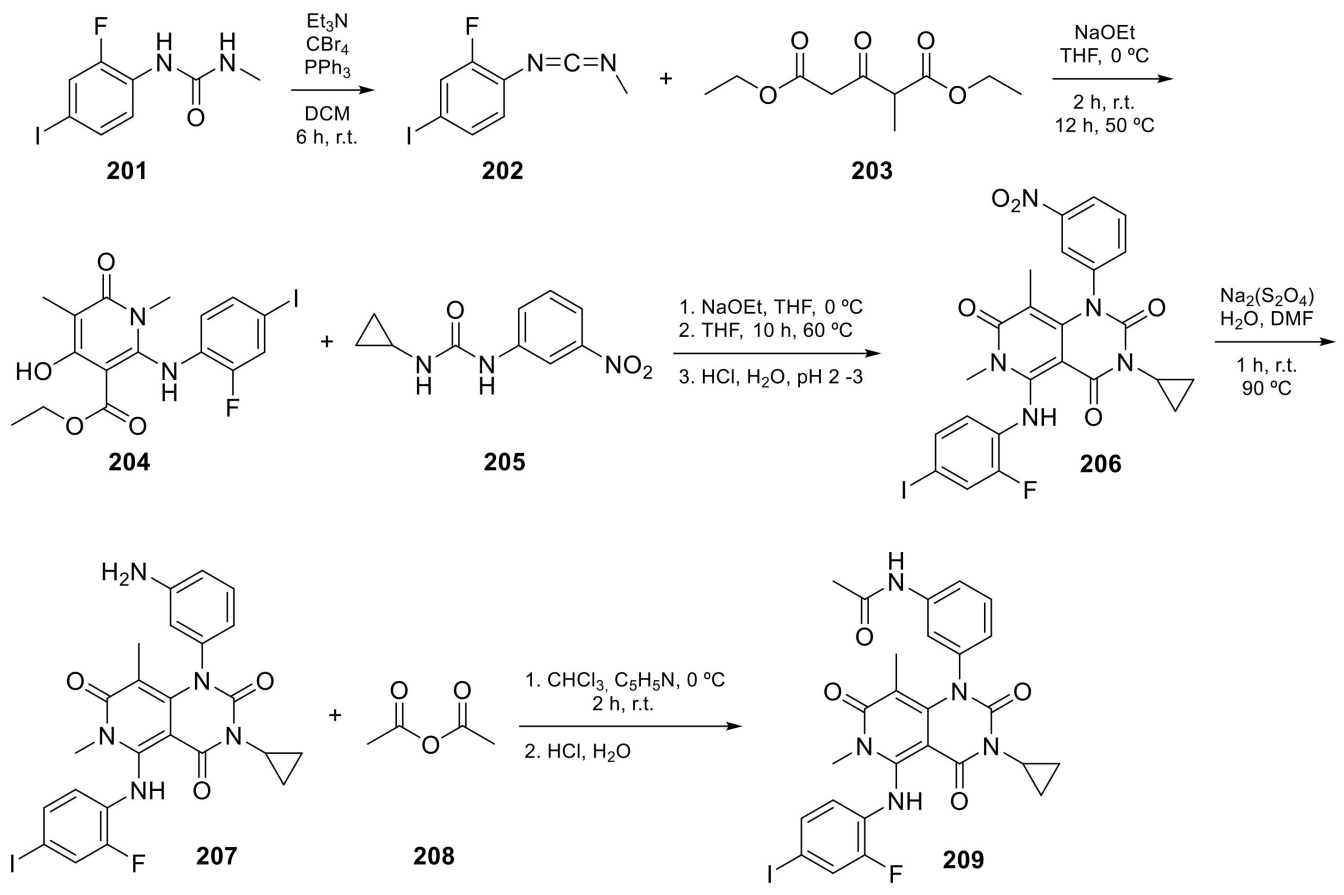
Synthesis of *N*-(3-(3-cyclopropyl-5-[(2-fluoro-4-iodophenyl)amino]-6,8-dimethyl-2,4,7-trioxo-1*H*,2*H*,3*H*,4*H*,6*H*,7*H*-pyrido[4,3-*d*]pyrimidin-1-yl)phenyl)acetamid, Trametinib (**209**) [69].

Trametinib (**209** in Scheme 21, Table 1, entry 21) is a kinase inhibitor used for specific types of melanoma. This compound, associated with other molecules such as Dabrafenib (Tafilnar) and/or Mekinist (trametinib), has been approved by the FDA in particular for the treatment of degenerative thyroid cancer (ATC) [66,67].

Trametinib has a dual specificity mitogen-activated protein kinase kinase 1/Dual specificity mitogen-activated protein kinase kinase 2 target.

MEK1 and MEK2 are dual-specificity kinases that activate ERK1 and ERK2 by phosphorylating them at preserved threonine and tyrosine residues in the T-E-Y motif found in their activation loop. MEK inhibitors are drugs that inhibit mitogen-activated protein kinase MEK1 and/or MEK2. They can be useful to influence the MAPK/ERK pathway, which is often intense in some types of cancer. MEK inhibitors bind to and inhibit MEK by inhibiting MEK-dependent cell signaling. This inhibition leads to cell death and tumor growth inhibition. They are allosteric inhibitors of MEK binding that inhibit either MEK1 itself or both MEK1 and MEK2 [68].

Shi et al. [69] patented the synthesis of Trametinib in 2019 as follows (Scheme 21).

### 2.4. Pyrido[3,2-d]pyrimidine

**Scheme 22:**
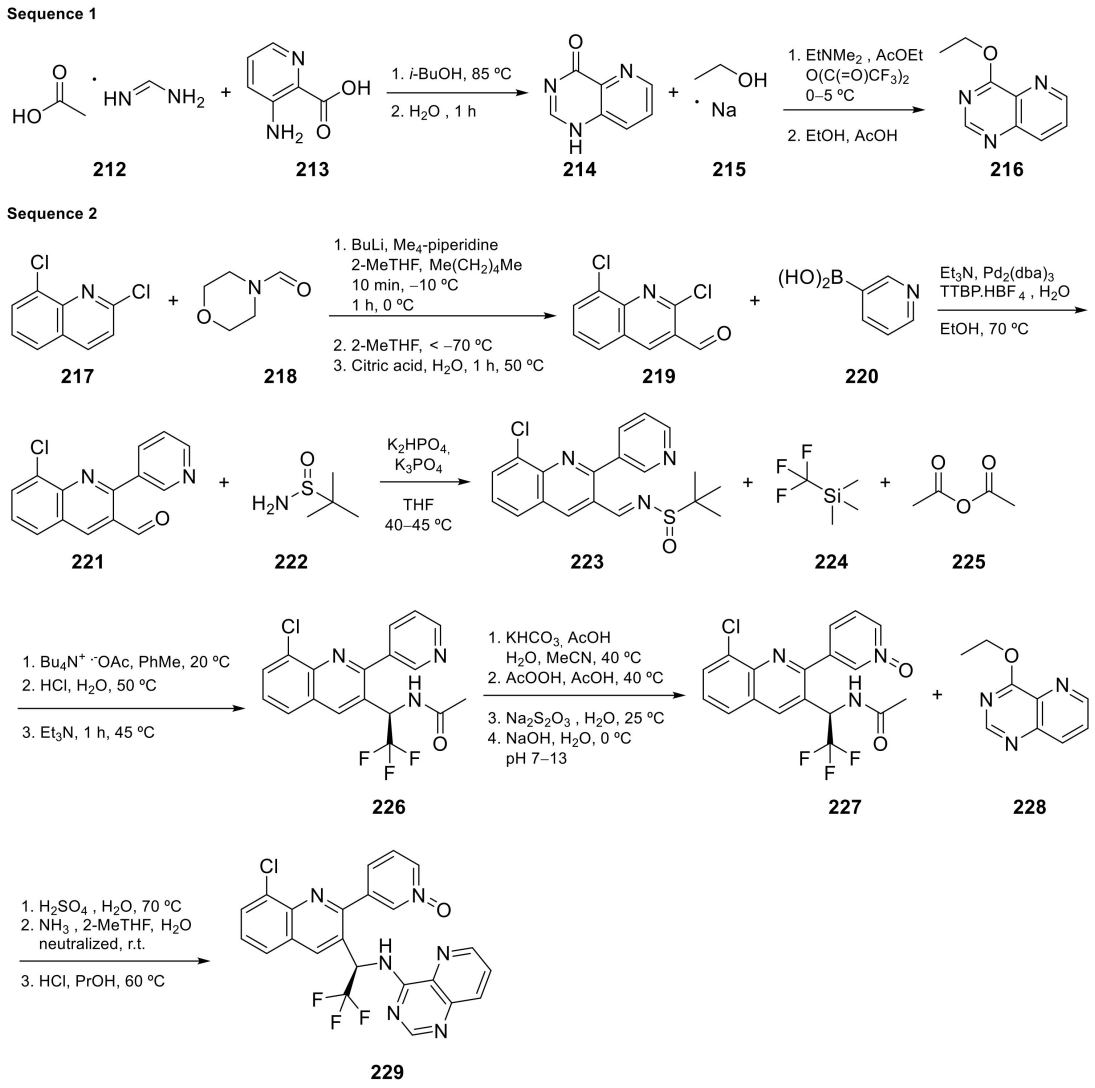
Synthesis of 3-[8-chloro-3-[(1*R*)-2,2,2-trifluoro-1-([pyrido[3,2-*d*]pyrimidin-4-yl]amino)ethyl]quinolin-2-yl]pyridin-1-ium-1-olate (**229**) [71].

Seletalisib (**229** in Scheme 22, Table 1, entry 22) is a novel small-molecule inhibitor of PI3Kδ that was evaluated in clinical assays to study the treatment and basic science of Primary Sjogren’s Syndrome [70]. This molecule is an ATP-competitive and highly selective PI3Kδ inhibitor. Phosphoinositide 3-kinases (PI3K) are enzymes regulating cellular survival, development, and function. They play a key role in immune cell development and function.

Le Meur et al. [71] patented the synthesis of Seletalisib **229** following the procedure summarized in Scheme 22, and described crystalline forms for the treatment of various pathologies.

**Scheme 23:**
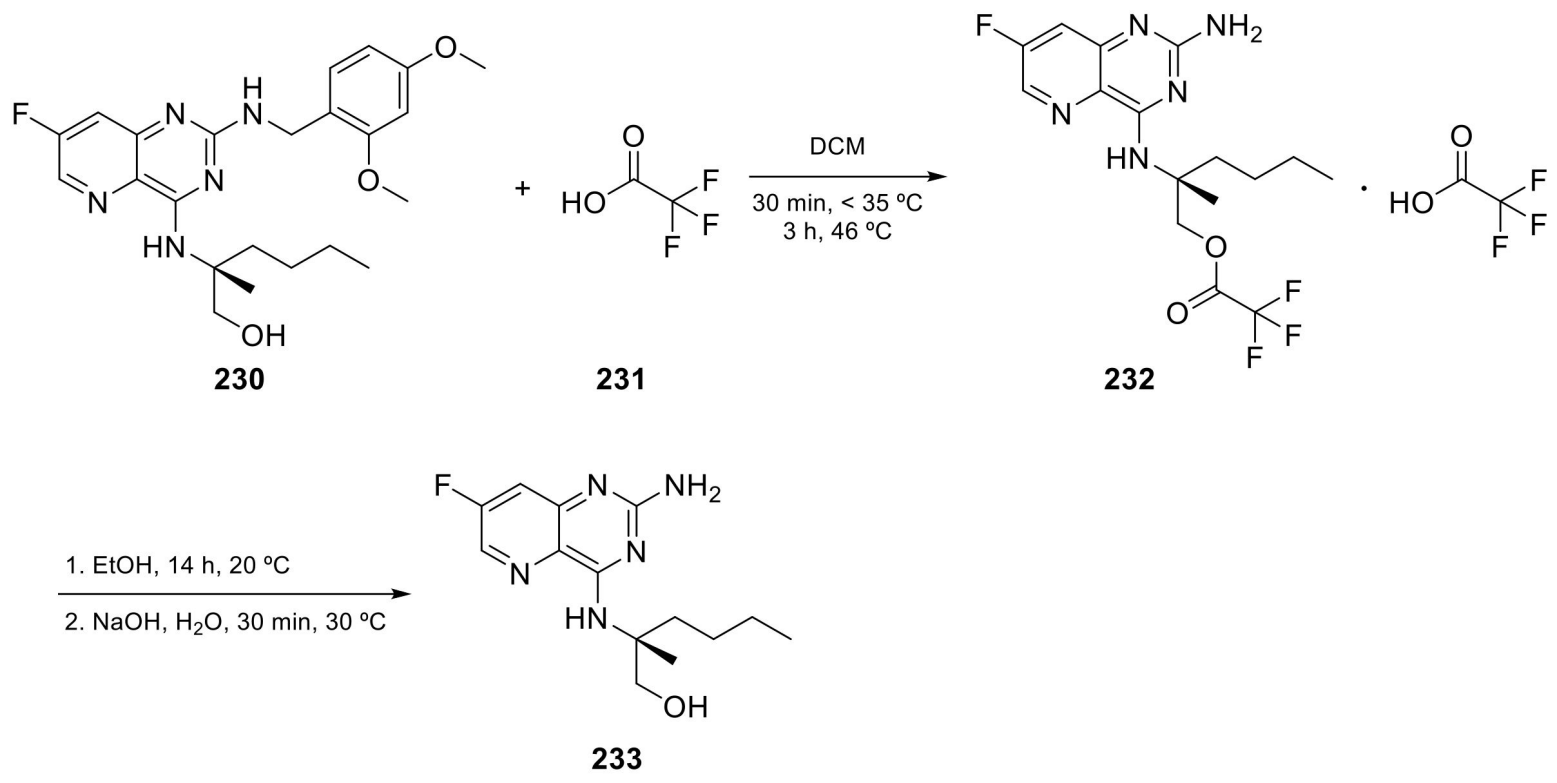
(2*S*)-2-([2-amino-7-fluoropyrido[3,2-*d*]pyrimidin-4-yl]amino)-2-methylhexan-1-ol (**233**) [73].

In the clinical trial of safety, tolerability and antiviral activity in virally suppressed adults with chronic hepatitis B, Selgantolimod **233** (Scheme 23, Table 1, entry 23) is being studied [72]. Chronic hepatitis B (CHB) is associated with a dysfunction of the immune response implicating toll-like receptor 8 (TLR8). Therefore, synthesizing a selective TLR8 agonist could be an effective treatment option [72].

Vieira et al. patented in 2020 the preparation of solid forms of (*R*)-2-[(2-amino-7-fluoropyrido[3,2-*d*]pyrimidin-4-yl)amino]-2-methylhexan-1-ol (**233**) as toll-like receptor modulators, as described in Scheme 23 [73].

**Scheme 24:**
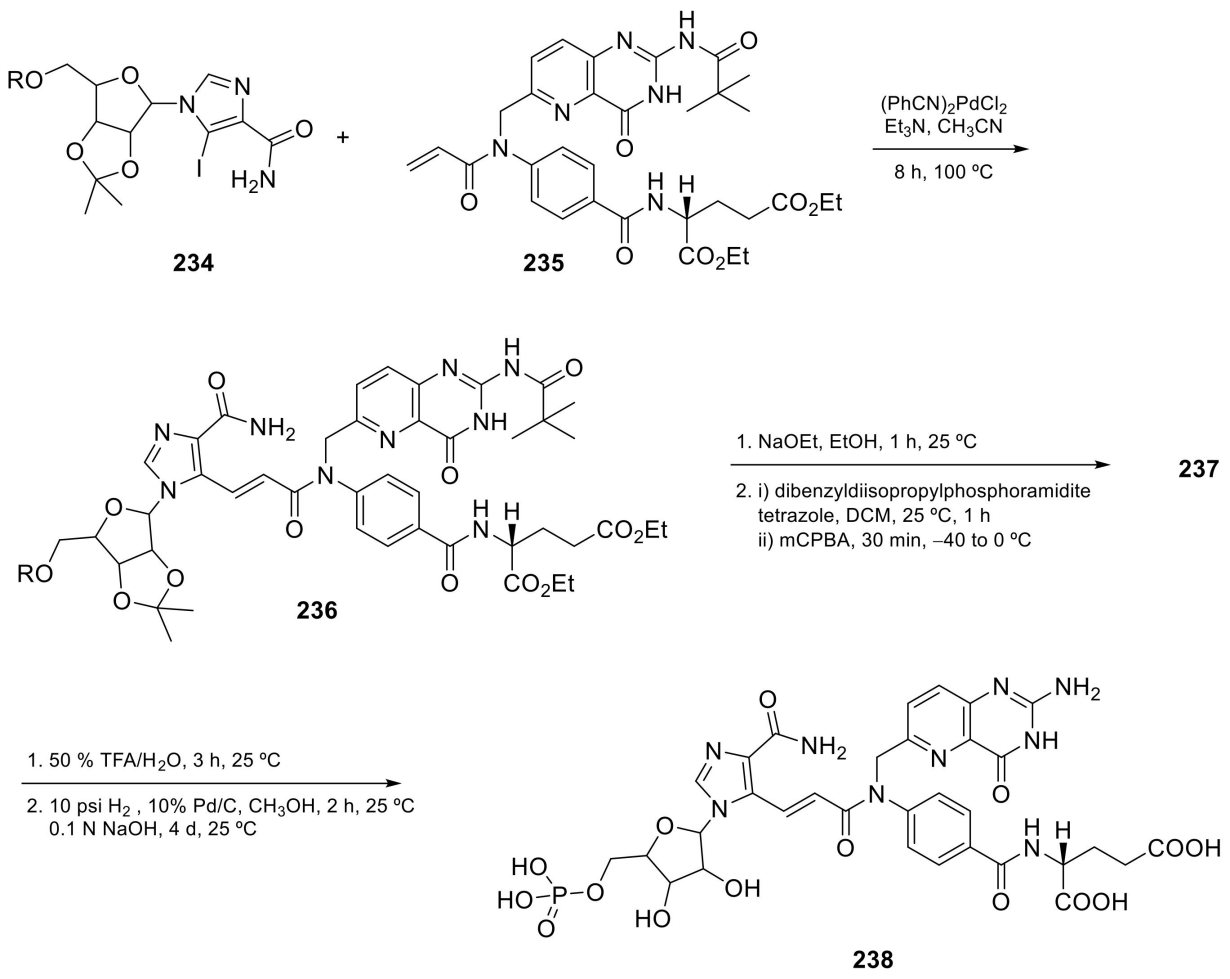
Synthesis of (2*S*)-2-((4-[(2*E*)-*N*-((2-amino-4-oxo-1*H*,4*H*-pyrido[3,2-*d*]pyrimidin-6-yl)methyl)-3-{4-carbamoyl-1-[(2*R*,3*R*,4*S*,5*R*)-3,4-dihydroxy-5-[(phosphonooxy)methyl]oxolan-2-yl]-1*H*-imidazol-5-yl)prop-2-enamido]phenyl)formamido)pentanedioic acid (**238**) [74].

Benkovic et al. presented the synthesis of β-DADF (**238** in Scheme 24, Table 1, entry 24) through an approach focused on a coupling reaction of the appropriately protected 5-iodoimidazole nucleoside **234** and 10-acryloyl folate derivative **235**, catalyzed by bis(benzonitrile)palladium chloride to afford compound **237**. 5′-Acetate was removed with sodium ethoxide, and 5′-OH phosphorylation was completed performing phosphoramidite procedures to obtain **238**. Acetonide removal was achieved with 50% TFA/H_2_O, followed by debenzylation with H_2_ on 10% Pd/C. The simultaneous saponification of ethyl and pivalate esters with 0.1 N NaOH finally gave β-DADF **239** [74]. This compound targets the bifunctional purine biosynthesis protein PURH, an enzyme catalyzing the last two steps in de novo purine biosynthesis [75].

All compounds described herein with their target were listed below (Table 1).

**Table 1:** The 24 pyridopyrimidines described in the review.

Entry	Structure	Name	Target	Ref.
Pyrido[2,3-*d*]pyrimidine				
1	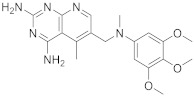	5-methyl-6-([methyl(3,4,5-trimethoxyphenyl)amino]methyl)pyrido[2,3-*d*]pyrimidine-2,4-diamine	DHFR Dihydrofolate reductase	[4,5,6]
2	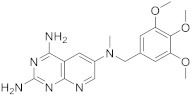	*N*6-methyl-*N*6-[(3,4,5-trimethoxyphenyl)methyl]pyrido[2,3-*d*]pyrimidine-2,4,6-triamine	DHFR Dihydrofolate reductase	[19]
3	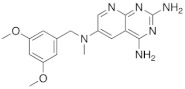	*N*6-[(3,5-dimethoxyphenyl)methyl]-*N*6-methylpyrido[2,3-*d*]pyrimidine-2,4,6-triamine,	DHFR Dihydrofolate reductase	[19]
4	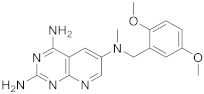	*N*6-[(2,5-dimethoxyphenyl)methyl]-*N*6-methylpyrido[2,3-*d*]pyrimidine-2,4,6-triamine	DHFR Dihydrofolate reductase	[18]
5	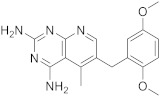 PIRITREXIM	6-[(2,5-dimethoxyphenyl)methyl]-5-methylpyrido[2,3-*d*]pyrimidine-2,4-diamine	DHFR Dihydrofolate reductase	[20,21,24]
6	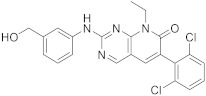	6-(2,6-dichlorophenyl)-2-([3-(hydroxymethyl)phenyl]amino)-8-ethyl-7*H*,8*H*-pyrido[2,3-*d*]pyrimidin-7-one	Tyrosine kinase activity	[25,26]
7	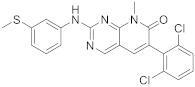 PD-173955	6-(2,6-dichlorophenyl)-8-methyl-2-([3-(methylsulfanyl)phenyl]amino)-7*H*,8*H*-pyrido[2,3-*d*]pyrimidin-7-one	Kinase activity: Tyrosine-protein kinase transforming protein Abl	[17]
8	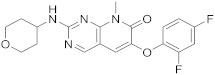	6-(2,4-difluorophenoxy)-8-methyl-2-[(oxan-4-yl)amino]-7*H*,8*H*-pyrido[2,3-*d*]pyrimidin-7-one	Kinase activity: Mitogen-activated protein kinase 14	[27,28,29]
9	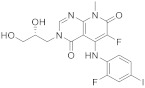 TAK-733	3-[(2*R*)-2,3-dihydroxypropyl]-6-fluoro-5-[(2-fluoro-4-iodophenyl)amino]-8-methyl-3*H*,4*H*,7*H*,8*H*-pyrido[2,3-*d*]pyrimidine-4,7-dione	Kinase activity: Against MEK and ERK	[30,31,32,33]
10	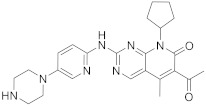 Palbociclib	6-acetyl-8-cyclopentyl-5-methyl-2-([5-(piperazin-1-yl)pyridin-2-yl]amino)-7*H*,8*H*-pyrido[2,3-*d*]pyrimidin-7-one	Kinase activity: Cyclin-dependent kinase 4/Cyclin-dependent kinase 6 Breast cancer drug	[34,35,36,37,38]
11	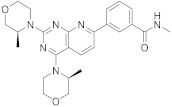 Vistusertib	3-(2,4-bis[(3*S*)-3-methylmorpholin-4-yl]pyrido[2,3-*d*]pyrimidin-7-yl)-*N*-methylbenzamide	Kinase activity: Vistusertib (AZD2014) is a novel mTOR inhibitor	[39,40]
12	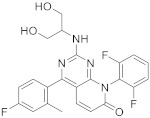 Dilmapimod (SB-681323)	8-(2,6-difluorophenyl)-2-[(1,3-dihydroxypropan-2-yl)amino]-4-(4-fluoro-2-methylphenyl)-7*H*,8*H*-pyrido[2,3-*d*]pyrimidin-7-one	Kinase activity: P38 MAPK inhibitor, Tumor necrosis factor/Interleukin-1 beta/Interleukin-6. Potential activity against rheumatoid arthritis	[41,42,43,44,45,46,47]
13	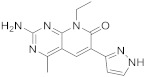 Voxtalisib	2-amino-8-ethyl-4-methyl-6-(1*H*-pyrazol-5-yl)-7*H*,8*H*-pyrido[2,3-*d*]pyrimidin-7-one	Kinase activity: PI3K/mTOR Inhibitor	[48,49]
14	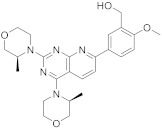 AZD8055	(5-(2,4-bis[(3*S*)-3-methylmorpholin-4-yl]pyrido[2,3-*d*]pyrimidin-7-yl)-2-methoxyphenyl)methanol	Kinase activity: Selective ATP-competitive mTOR kinase inhibitor. Induction of MEK/ERK	[50,51]
15	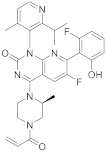 AMG-510	6-fluoro-7-(2-fluoro-6-hydroxyphenyl)-1-[4-methyl-2-(propan-2-yl)pyridin-3-yl]-4-[(2*S*)-2-methyl-4-(prop-2-enoyl)piperazin-1-yl]-1*H*,2*H*-pyrido[2,3-*d*]pyrimidin-2-one	Kinase Activity: KRAS inhibitor implicated in the RAS/MAPK pathway GTPase KRas	[52,53,54,55,56]
16	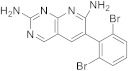	6-(2,6-dibromophenyl)pyrido[2,3-*d*]pyrimidine-2,7-diamine	Biotin carboxylase	[57]
17	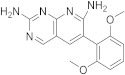	6-(2,6-dimethoxyphenyl)pyrido[2,3-*d*]pyrimidine-2,7-diamine	Biotin carboxylase	[25,58]
18	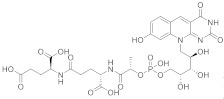	(2*S*)-2-[(4*S*)-4-carboxy-4-[(2*S*)-2-([hydroxy(([(2*R*,3*S*,4*S*)-2,3,4-trihydroxy-5-(8-hydroxy-2,4-dioxo-2*H*,3*H*,4*H*,10*H*-pyrimido[4,5-*b*]quinolin-10-yl)pentyl]oxy))phosphoryl]oxy)propanamido]butanamido]pentanedioic acid	Methanobacterium redox coenzyme Factor 420 (F_420_)	[59]
**Pyrido[3,4-*d*]pyrimidine**				
19	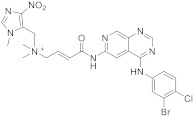 Tarloxotinib	[(2*E*)-3-((4-[(3-bromo-4-chlorophenyl)amino]pyrido[3,4-*d*]pyrimidin-6-yl)carbamoyl)prop-2-en-1-yl]dimethyl[(1-methyl-4-nitro-1*H*-imidazol-5-yl)methyl]azanium	Kinase Activity: Pan-HER kinase inibitor	[61]
20	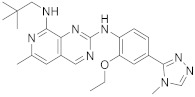 BOS172722	*N*8-(2,2-dimethylpropyl)-*N*2-[2-ethoxy-4-(4-methyl-4*H*-1,2,4-triazol-3-yl)phenyl]-6-methylpyrido[3,4-*d*]pyrimidine-2,8-diamine	Kinase Activity: Dual specificity protein kinase TTK	[62,63,64,65]
**Pyrido[4,3-*d*]pyrimidine**				
21	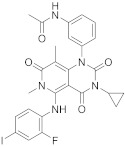 Trametinib	*N*-(3-(3-cyclopropyl-5-[(2-fluoro-4-iodophenyl)amino]-6,8-dimethyl-2,4,7-trioxo-1*H*,2*H*,3*H*,4*H*,6*H*,7*H*-pyrido[4,3-*d*]pyrimidin-1-yl)phenyl)acetamide	Dual specificity mitogen-activated protein kinase kinase 1/Dual specificity mitogen-activated protein kinase kinase 2	[66,67,68,69]
**Pyrido[3,2-*d*]pyrimidine**				
22	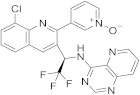 Seletalisib	3-(8-chloro-3-[(1*R*)-2,2,2-trifluoro-1-((pyrido[3,2-*d*]pyrimidin-4-yl)amino)ethyl]quinolin-2-yl)pyridin-1-ium-1-olate	selective PI3Kδ inhibitor	[70,71]
23	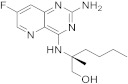	(2*S*)-2-((2-amino-7-fluoropyrido[3,2-*d*]pyrimidin-4-yl)amino)-2-methylhexan-1-ol	Chronic hepatitis B TLR8 receptor	[72,73]
24	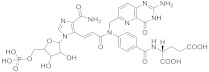 β-DADF	(2*S*)-2-((4-[(2*E*)-*N*-((2-amino-4-oxo-1*H*,4*H*-pyrido[3,2-*d*]pyrimidin-6-yl)methyl)-3-(4-carbamoyl-1-[(2*R*,3*R*,4*S*,5*R*)-3,4-dihydroxy-5-[(phosphonooxy)methyl]oxolan-2-yl]-1*H*-imidazol-5-yl)prop-2-enamido]phenyl)formamido)pentanedioic acid	Bifunctional purine biosynthesis protein PURH	[74,75]

## 3. Conclusions

Herein we have summarized some pyridopyrimidines (Table 1) that have a real therapeutic potential. Their biological activity and synthetic pathways are described. This review shows the interest of this heterocycle family and its high representativity in the drugs on the market or in the process of being marketed. It is, therefore, always interesting to highlight new access routes to these molecules of interest.
